# LSD1 activation promotes inducible EMT programs and modulates the tumour microenvironment in breast cancer

**DOI:** 10.1038/s41598-017-17913-x

**Published:** 2018-01-08

**Authors:** T. Boulding, R. D. McCuaig, A. Tan, K. Hardy, F. Wu, J. Dunn, M. Kalimutho, C. R. Sutton, J. K. Forwood, A. G. Bert, G. J. Goodall, L. Malik, D. Yip, J. E. Dahlstrom, A. Zafar, K. K. Khanna, S. Rao

**Affiliations:** 1 Health Research Institute, Faculty of ESTeM, University of Canberra, Bruce, ACT 2617 Australia; 2 QIMR Berghofer Medical Research Institute, 300 Herston Road, Herston, QLD 4006 Australia; 3 School of Biomedical Sciences, Charles Sturt University, Wagga Wagga, NSW 2678 Australia; 4 Gene Regulation Section, Centre for Cancer Biology, SA Pathology, Adelaide, SA 5000 Australia; 5 Department of Medical Oncology, The Canberra Hospital, Garran, ACT 2605 Australia; 6 ANU Medical School, Australian National University, Acton, ACT 2601 Australia; 7 Department of Anatomical Pathology, ACT Pathology, The Canberra Hospital, Garran, ACT 2605 Australia

**Keywords:** Breast cancer, Breast cancer, Cell biology, Cell biology

## Abstract

Complex regulatory networks control epithelial-to-mesenchymal transition (EMT) but the underlying epigenetic control is poorly understood. Lysine-specific demethylase 1 (LSD1) is a key histone demethylase that alters the epigenetic landscape. Here we explored the role of LSD1 in global epigenetic regulation of EMT, cancer stem cells (CSCs), the tumour microenvironment, and therapeutic resistance in breast cancer. LSD1 induced pan-genomic gene expression in networks implicated in EMT and selectively elicits gene expression programs in CSCs whilst repressing non-CSC programs. LSD1 phosphorylation at serine-111 (LSD1-s111p) by chromatin anchored protein kinase C-theta (PKC-θ), is critical for its demethylase and EMT promoting activity and LSD1-s111p is enriched in chemoresistant cells *in vivo*. LSD1 couples to PKC-θ on the mesenchymal gene epigenetic template promotes LSD1-mediated gene induction. *In vivo*, chemotherapy reduced tumour volume, and when combined with an LSD1 inhibitor, abrogated the mesenchymal signature and promoted an innate, M1 macrophage-like tumouricidal immune response. Circulating tumour cells (CTCs) from metastatic breast cancer (MBC) patients were enriched with LSD1 and pharmacological blockade of LSD1 suppressed the mesenchymal and stem-like signature in these patient-derived CTCs. Overall, LSD1 inhibition may serve as a promising epigenetic adjuvant therapy to subvert its pleiotropic roles in breast cancer progression and treatment resistance.

## Introduction

Epithelial-to-mesenchymal transition (EMT) is a plastic cellular process in which fully differentiated epithelial cells reversibly dedifferentiate into cells with mesenchymal characteristics^1^. EMT is physiological in embryonic development and wound healing^2^ but pathological in cancer, where it endows cells with highly aggressive traits that facilitate dissemination, therapeutic resistance, and relapse^3^. EMT results in cytoskeletal restructuring, loss of cell-cell adhesion, and abnormal apical-basal polarity, thereby increasing motility and invasion of the cancer cells^1,2^. EMT also induces the formation of cancer stem cells (CSCs) with tumourigenic and metastatic capabilities^4^ that, although only constituting a small proportion of the main tumour bulk, possess the capacity to self-renew and differentiate^5^ and enhance resistance to chemotherapy, ionizing radiation, and hormone therapies^6^.

EMT is regulated by extensive modifications to the histone template and many hallmark changes during EMT are dependent on these alterations^7,8^. Histone methylation was originally thought to be fixed, but the discovery of the first histone demethylase, lysine-specific demethylase 1 (LSD1), highlighted that this modification was dynamic^9^. LSD1 is a chromatin-modifying enzyme that selectively catalyzes the removal of mono- and dimethylated groups from H3K4 and H3K9 depending on its association with nuclear receptors^10,11^. H3K4 methylation is generally associated with gene activation and H3K9 methylation with gene repression, so LSD1 acts as a dual transcriptional activator and repressor depending on its target residue^12^.

LSD1 is implicated in the pathogenesis of various epithelial cancers in the prostate, bladder, liver, non-small cell lung cancer (NSCLC), and neuroblastomas^13–18^. In breast cancer, LSD1 expression increases with progression from ductal carcinomas *in situ* (DCIS) to invasive ductal carcinoma^19^ and overexpression is positively correlated with estrogen receptor negative (ER^−^) status^20^. Knockdown or inhibition of LSD1 reduces both the invasiveness and proliferative capacity of breast cancer cells *in vitro* ^21,22^.

LSD1 is implicated in the regulation of EMT. Specifically, LSD1 mediates global EMT-related epigenetic reprogramming in mouse hepatocytes, thereby facilitating cell migration and chemoresistance^23^, and LSD1 promotes EMT, migration, and proliferation in NSCLC and hepatocellular carcinoma^16,18^. Several physiological events occur during EMT including loss of the epithelial transmembrane protein E-cadherin (encoded by *CDH1*)^24^ mediated by EMT-inducing transcription factors (EMT-TFs) including Snail, Slug and Zinc finger E-box-binding homeobox 1 (ZEB1)^25,26^. In breast cancer, Snail recruits LSD1 to epithelial gene promoters including that of *CDH1*, where LSD1 mediates H3K4me2 demethylation and consequent transcriptional silencing^27,28^. Additionally, LSD1 can interact with Slug to promote migration and invasion of breast cancer cell lines^29,30^. Recent studies have implicated LSD1 in breast CSC self-renewal^31^. However, the global epigenetic effects of LSD1 in breast cancer cells, the tumour microenvironment, and therapeutic resistance are unknown.

Somatic mutations in tumour can result in dysregulation of an inflammatory response and induce a pro-tumour environment^32^. Notably, recent studies have suggested that tumour-associated stroma also builds a supportive environment for tumour cell proliferation, invasion, dissemination and immune evasion^33^. Tumour stroma comprises heterogeneous collection of cells, amongst this cancer-associated fibroblasts (CAFs), macrophages and myeloid-derived cells promote tumourigenesis through paracrine mechanisms in particular through the secretion of cytokines and growth factors^34^. Moreover, CAFs have emerged as important participants in cancer progression and are implicated in regulating CSC self-renewal in the tumour niche^35^. CAF induction can be characterised by an increase in fibroblast activation protein (FAP) as well as CAF-induced cytokine *CCL2* ^35,36^.

Here we evaluated the role of LSD1 in the global epigenetic regulation of EMT, CSCs, and therapeutic resistance in breast cancer. We show that LSD1 regulates several breast cancer programs, namely EMT, CSC formation and tumour progression. Pharmacological inhibition of LSD1 in combination with existing chemotherapy regimens effectively reduces chemotherapy-induced EMT, and CAF burden whilst promoting innate M1 macrophage infiltration at the primary tumour site. Inhibition of LSD1 in CTCs isolated from patients with MBC successfully reduces the mesenchymal and stem-like burden of these cells. Thus, LSD1 is an exemplar epigenetic target in breast cancer.

## Results

### LSD1 is induced during EMT and regulates key EMT markers

**Figure 1:**
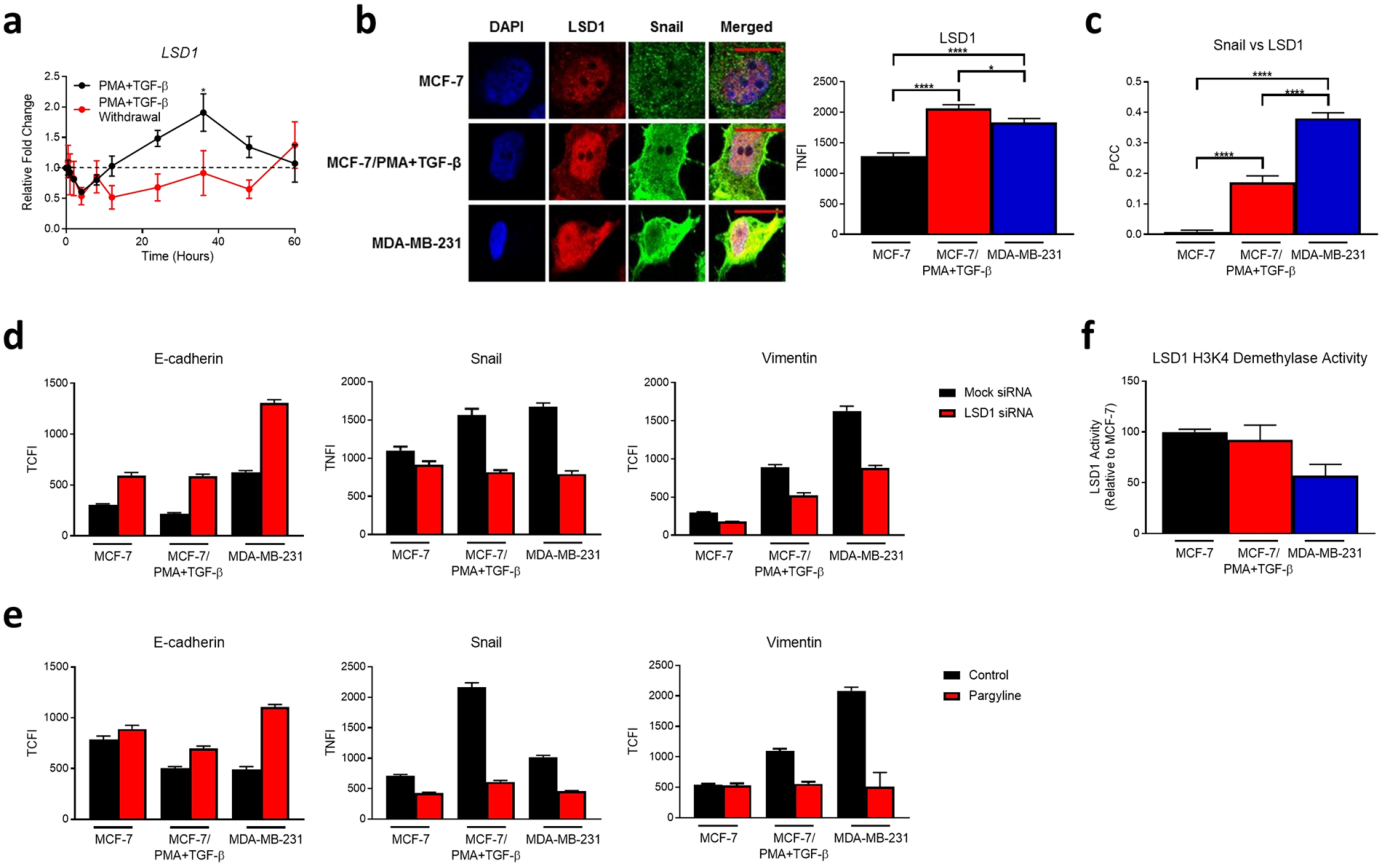
LSD1 is induced in mesenchymal cells and promotes breast cancer EMT markers. (**a**) *LSD1* transcript levels measured by qPCR in MCF-7 cells after incubation with PMA + TGF-β or withdrawal of PMA+TGF-β after 60 h incubation at the indicated time points. Data are expressed as fold change relative to 0 h stimulation or 0 h stimulation withdrawal (n = 3). *Indicates significance relative to 0 h stimulation. (**b**) Immunofluorescence microscopy was performed on cells fixed and probed with primary anti-LSD1 or anti-Snail antibodies and DAPI. Representative images for each dataset are shown. Graph represents the TNFI values for LSD1 measured using ImageJ to select the nucleus minus background (n > 50 individual cells). (**c**) The PCC was determined for LSD1 and Snail (n = 40 individual cells). −1 = inverse of colocalization; 0 = no colocalization; +1 = perfect colocalization. TCFI of E-cadherin and vimentin and TNFI of Snail and in MCF-7, MCF-7/PMA+ TGF-β and MDA-MB-231 cells after treatment with either (**d**) mock and LSD1 siRNA; or (**e**) vehicle alone and pargyline (n ≥ 30 individual cells). (**f**) LSD1 H3K4 demethylation activity assay was performed on MCF-7, MCF-7/PMA+ TGF-β, and MDA-MB-231 nuclear extracts in triplicate wells. LSD1 demethylase activity was calculated and graph depicts percentage of activity relative to MCF-7 cells (n = 2). All data represents the mean ±SE. Scale bars = 10 µm. *P < 0.05; **P < 0.01; ***P < 0.001; ****P < 0.0001, Mann-Whitney test.

To understand how LSD1 functions in EMT, epithelial MCF-7 cells were treated with the well-established EMT-inducers phorbol-12-myristate-13-acetate (PMA) and transforming growth factor-β (TGF-β)^4,37,38^, which potentiate EMT in MCF-7 cells when administered in combination^38^. EMT was confirmed by the hallmark loss of E-cadherin and induction of vimentin and Snail (Supplementary Fig. 1a,b), as well as phenotypic morphological changes as early as 2 hours after treatment with PMA + TGF-β (Supplementary Fig. 1c). *LSD1* expression peaked at 36 hours and then returned to baseline after 60 hours of treatment with PMA + TGF-β, and, after PMA + TGF-β withdrawal at 60 hours, *LSD1* expression decreased before returning to near basal levels indicating that *LSD1* induction is reversible (Fig. 1a). Moreover, the spatiotemporal induction pattern of *LSD1* was largely consistent with that observed for the key EMT-TF, *Snail* from as early as 24 hours during both PMA + TGF-β-treatment and after PMA + TGF-β withdrawal (Supplementary Fig. 1d). At the protein level immunofluorescence microscopy revealed a significant increase in LSD1 total nuclear fluorescence intensity (TNFI) following induction of EMT in MCF-7/PMA + TGF-β cells and in fully dedifferentiated mesenchymal MDA-MB-231 cells (Fig. 1b). Additionally, induction of EMT was observed in both T47D and ZR751 cells after PMA + TGF-β (Supplementary Fig. 1e), which was accompanied by an induction of LSD1 at the protein level (Supplementary Fig. 1f). Given that LSD1 has previously been shown to interact with Snail *in vitro* ^28^, we examined their relationship and whether it differed in the mesenchymal state. Using the Pearson’s correlation co-efficient (PCC) of Snail and LSD1 as a measure of colocalization, we found a significant increase in their colocalization in MCF-7/PMA + TGF-β cells (PCC = 0.170) and MDA-MB-231 cells (PCC = 0.380) (Fig. 1c).

As these data indicate that LSD1 is induced during EMT, we were interested in examining LSD1 expression after inducing the opposing biological process, mesenchymal-to-epithelial transition (MET). To do so, mesenchymal MDA-MB-231, MDA-MB-436 and SUM149 PT cells were treated with erlotinib, which has previously been identified as a MET-inducer. MET was identified in each cell line by a dose-dependent increase in E-cadherin, and reduction in vimentin, Snail and ZEB1 (Supplementary Fig. 2a). Interestingly, we observed a dose-dependent reduction in LSD1 protein levels after MET induction in all cell lines (Supplementary Fig. 2b). Moreover, MET was induced in MDA-MB-231 cells using either compound 27 (C27), a specific PKC-θ inhibitor, or bisindolylmaleimide I (BIM), a pan-PKC inhibitor (Supplementary Fig. 2c). Again, we observed a reduction in LSD1 protein levels after MET induction (Supplementary Fig. 2d). Collectively, these results suggest that LSD1 expression is proportional to the mesenchymal status of breast cancer cells.

Since LSD1 may, therefore, regulate downstream events during EMT, we examined several key EMT markers after treatment with pargyline (an LSD1 and its monoamine oxidase activity inhibitor^39^) or a previously validated pool of three specific 19–25-nucleotide LSD1 small interfering RNAs (siRNAs) targeting LSD1 (knockdown confirmed by microscopy and qPCR; Supplementary Fig. 3). LSD1 inhibition increased E-cadherin total cell fluorescence intensity (TCFI) and decreased Snail TNFI and vimentin TCFI in MCF-7, MCF-7/PMA + TGF-β, and MDA-MB-231 cells (Fig. 1d). Similar results were observed after siRNA-mediated knockdown of LSD1 in MCF-7, MCF-7/PMA + TGF-β and MDA-MB-231 cells (Fig. 1e). To further explore the role of LSD1 during EMT, we examined LSD1 activity based on its demethylation of H3K4 residues. LSD1 activity was 45% lower in MDA-MB-231 cells than MCF-7 cells (Fig. 1f), suggesting that LSD1 is more repressive in luminal type epithelial MCF-7 cells than basal type mesenchymal MDA-MB-231 cells. Collectively, as reported previously, LSD1 modulates breast cancer EMT, colocalises with the key EMT-TF Snail, and regulates the expression of several hallmark EMT proteins including E-cadherin and vimentin. Notably, we report here reduced repressive activity of LSD1 in mesenchymal cells.

### LSD1 tethers to chromatin and regulates primary and secondary genome-wide programs that activate EMT-related pathways

**Figure 2:**
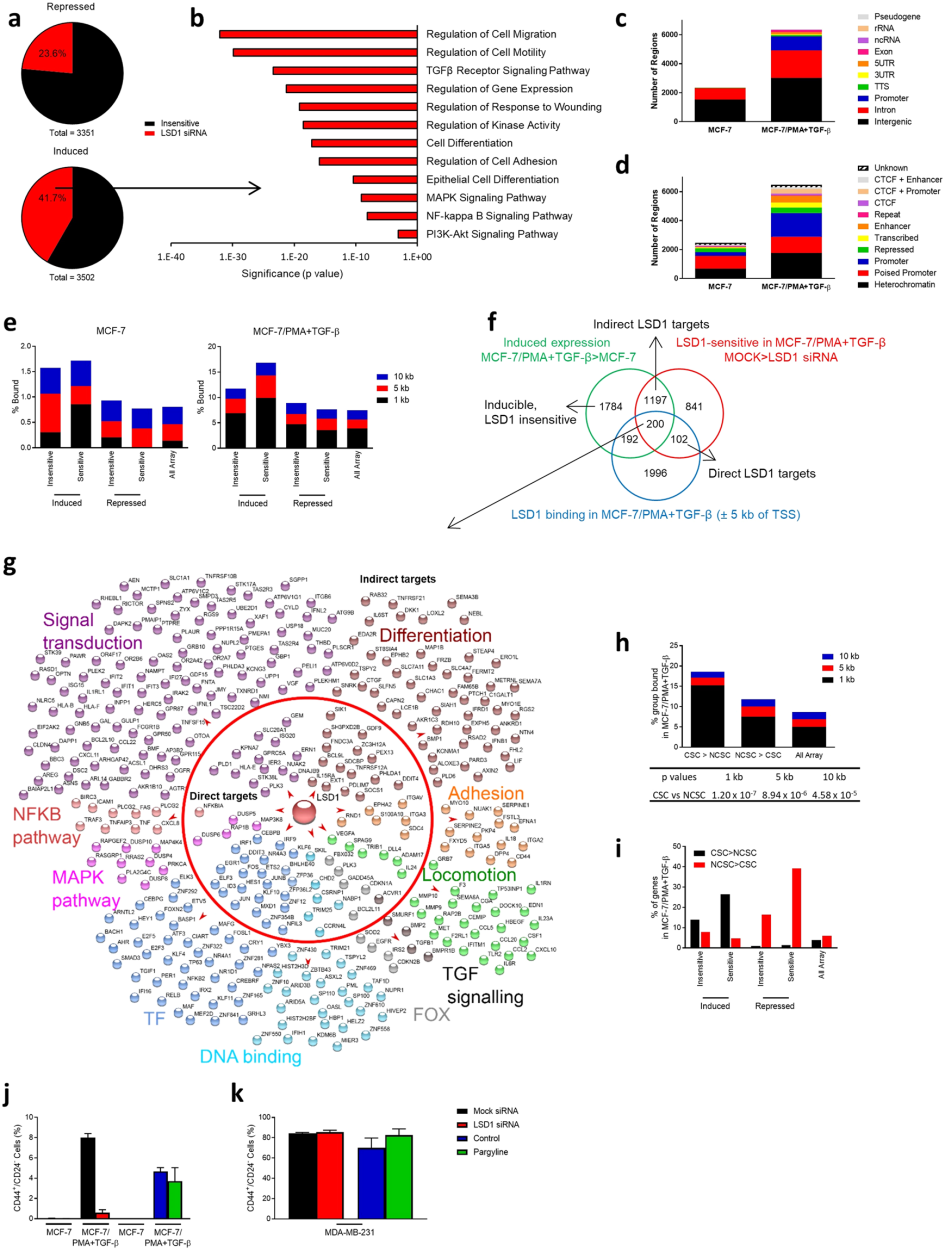
LSD1 selectively targets gene induction programs promoting EMT and CSCs. (**a**) Induced and repressed probes following treatment with either mock or LSD1 siRNA in MCF-7/PMA+ TGF-β cells. Probes with a log_2_ (0.5)-fold difference were considered induced or repressed. (**b**) Induced LSD1-sensitive genes were profiled for enrichment of biological processes and KEGG pathways. The significance of the enrichment of each group is indicated, hypergeometric test. LSD1 ChIP-seq peaks were annotated to: (**c**) summarised chromatin environments of the nearest Ensembl transcript. Peaks were classified as rRNA, 5′UTR, promoter (<1 kb from TSS), intergenic, intron, exon, pseudo, TTS, ncRNA, or 3′UTR; and (**d**) summarised CTCF/chromatin states in MCF-7 cells. (**e**) LSD1 ChIP-seq peaks were grouped by indicated distance to the nearest TSS of induced or repressed genes based on their sensitivity to LSD1 siRNA in MCF-7 and MCF-7/PMA+ TGF-β cells. Graphs represent the percentage of genes that lie within each group. (**f**) Venn diagram depicting the separation of LSD1 ChIP-seq peaks in MCF-7/PMA+ TGF-β cells. (**g**) Induced LSD1-sensitive genes with GO or KEGG annotations in indicated pathways. Genes within the circle are direct LSD1 targets, outside the circle are indirect LSD1 targets. (**h**) LSD1 ChIP-seq-peaks were annotated by indicated distance to the nearest TSS of CSC > NCSC or NCSC > CSC transcripts in MCF-7/PMA+ TGF-β cells. Graphs represent the percentage of genes that lie within each group. Fisher’s exact t test was performed comparing CSCs vs NCSCs and p values are shown for indicated distances. (**i**) Percent of genes that are LSD1 sensitive or LSD1 insensitive and either expressed CSC > NCSC or NCSC > CSC. Percentage CD44^+^/CD24^−^ cells as measured by flow cytometry after treatment with vehicle alone, pargyline, mock siRNA or LSD1 siRNA in (**j**) MCF-7 and MCF-7/PMA+ TGF-β; or (**k**) MDA-MB-231 cells ±SE (n = 3).

To further elucidate LSD1’s contribution to breast cancer EMT, we performed RNA expression profiling using Affymetrix microarrays on MCF-7 cells transfected with either LSD1 or mock siRNAs after PMA + TGF-β treatment. Of the 3502 probes upregulated during EMT, 1459 (41.7%) were reversible on LSD1 siRNA knockdown and, of the 3351 probes downregulated during EMT, 790 (23.6%) were reversible (Fig. 2a). The induced LSD1-sensitive genes were enriched for EMT-related processes including cell migration, cell motility, response to wounding, cell differentiation, and cell adhesion (Fig. 2b).

To help determine which LSD1-sensitive genes were direct LSD1 targets, we performed Chromatin immunoprecipitation-sequencing (ChIP-seq) on MCF-7 and MCF-7/PMA + TGF-β cells. Considerably more LSD1 binding regions (6487) were detected in PMA + TGF-β cells. LSD1 largely occupied intergenic and intronic regions in MCF-7 and MCF-7/PMA + TGF-β cells, and there was a substantial increase in the proportion of LSD1 bound to regions annotated as promoters during EMT (Fig. 2c). Utilizing information on the chromatin state and CCCTC-binding factor (CTCF) binding in these regions in MCF-7 cells^40^, we found that a large proportion of the MCF-7/PMA + TGF-β regions were marked as active promoters (as defined by H3K4me3 and H3K27ac) (Fig. 2d).

We overlaid our MCF-7 and MCF-7/PMA + TGF-β gene expression sets with LSD1 ChIP-seq data to determine if LSD1 preferentially tethered to induced or repressed genes, and within what distance of the TSSs of differentially expressed genes. After EMT induction, LSD1 occupied a higher proportion of regions within all distances of an induced gene transcription start site (TSS) than a repressed gene TSS (Fig. 2e). Furthermore, a greater proportion of the LSD1-sensitive induced genes had LSD1 binding than repressed genes (Fig. 2e). However, only around 17% of genes induced during EMT had chromatin-tethered LSD1 within 10 kb of the TSS. DNA looping enables distant binding of factors, and up to 39% of the LSD1-sensitive induced genes had LSD1 binding within 100 kb. It is, therefore, likely that a large proportion of LSD1-sensitive genes are induced due to indirect effects of LSD1. As a conservative approach, we classified those genes with LSD1-sensitive gene expression and LSD1 binding within 5 kb of their TSS as direct LSD1 targets and those without as indirect (Fig. 2f).

We next analyzed all directly and indirectly targeted LSD1-sensitive genes and classified them according to EMT-related pathways. Several genes in each group were both direct and indirect LSD1 targets, indicating a broad role for this enzyme in promoting EMT (Fig. 2g). The ratio of indirect to direct LSD1 targets was slightly lower for TF and forkhead box (FOX) proteins compared to other groups, suggesting that LSD1 may preferentially target TFs to achieve its secondary effects. Indeed, our recent study^41^ on the regulation of DNA accessibility in MCF-7/PMA cells identified an important role for AP-1 family members such as JUN, JUNB, and FOS, which are direct LSD1 targets (Fig. 2g). Therefore, LSD1 executes genome-wide EMT programs through both direct and indirect actions, the latter most likely via its target TFs.

### LSD1 selectively targets CSCs during EMT

Given that EMT is known to induce CSC formation, we next investigated whether LSD1 regulates breast CSCs. We overlaid LSD1 ChIP-seq and expression data with our previously published^38^ expression profiles generated from CSCs and non-CSCs (NCSCs) to explore the role of LSD1 in CSC regulation. A significantly higher proportion of LSD1-bound regions in MCF-7/PMA + TGF-β cells lay closer to genes induced in CSCs compared to NCSCs (Fig. 2h). We next intersected the LSD1 siRNA expression profiles with the CSC and NCSC expression profiles to determine any differential expression of these genes between the two cell subsets. A significantly (p = 1.3 × 10^−9^) greater proportion of the induced LSD1-sensitive genes had higher expression in CSCs than NCSCs compared to the inducible LSD1-insensitive genes. Furthermore, the repressed LSD1-sensitive genes had significantly (p < 2.2 × 10^−16^) biased expression toward NCSCs compared to the repressed LSD1-insensitive genes (Fig. 2i).

To validate any phenotypic correlations *in vitro*, we next examined the CD44^+^/CD24^−^ breast CSC composition in MCF-7, MCF-7/PMA + TGF-β and MDA-MB-231 cells. Treatment with LSD1 siRNA nearly completely abrogated CD44^+^/CD24^−^ CSC formation after PMA + TGF-β-induced EMT in MCF-7 cells, whilst pargyline was partially inhibitory (Fig. 2j). These results are consistent with previous reports implicating LSD1 in breast CSC regulation^31^. However, in MDA-MB-231 cells with a high constitutive CSC population^42^, LSD1 knockdown had no effect on their maintenance whilst LSD1 inhibition promoted their formation (Fig. 2k). These differences between MCF-7/PMA + TGF-β cells and MDA-MB-231 cells are likely to be in part due to differential roles for LSD1 in the formation compared to the maintenance, of breast CSCs. Nevertheless, our data indicates that LSD1 selectively targets and promotes breast CSCs by regulating inducible gene expression programs whilst acting as a repressor of NCSCs.

### PKC-θ directly phosphorylates LSD1 at serine-111 and regulates its repressive activity and nuclear localization

**Figure 3:**
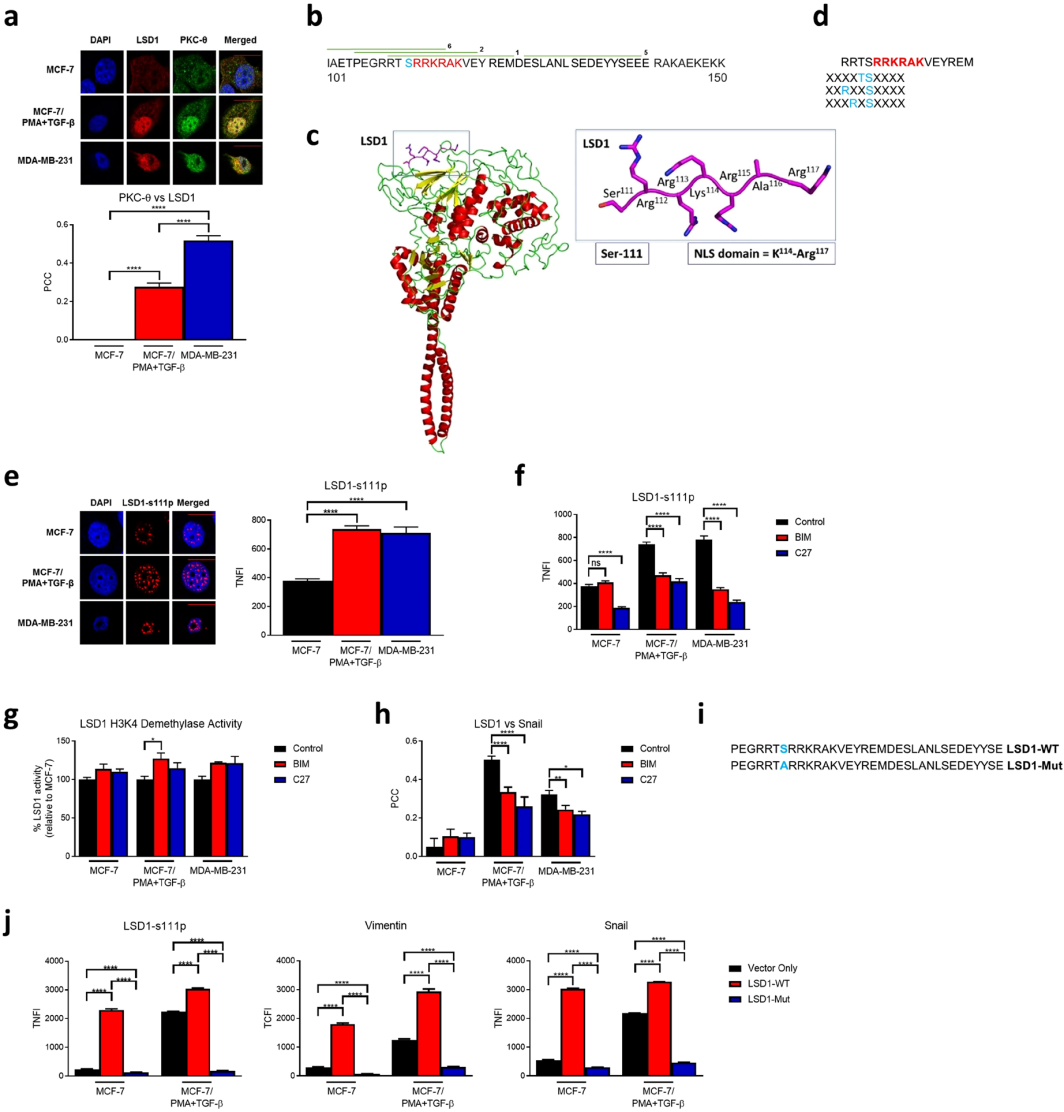
Phosphorylation of LSD1 at serine-111 by PKC-θ regulates its pro-EMT function. (**a**) Immunofluorescence microscopy was performed on indicated cells fixed and probed with anti-LSD1, anti-PKC-θ and DAPI. Representative images for each dataset are shown. Graph depicts the PCC for LSD1 and PKC-θ (n = 30 individual cells). −1 = inverse of colocalization; 0 = no colocalization; +1 = perfect colocalization. (**b**) Partial LSD1 amino acid sequence indicating the location of peptides positive for phosphorylation. Red amino acids = NLS region; blue amino acid = serine-111; green bars = location of peptides and are numbered in order of mean signal intensity. (**c**) Model of LSD1 generated using Phyre2, highlighting the close proximity of the serine-111 phosphorylation site to the positively charged NLS domain (inner box). LSD1 structure is based on PDB code 2V1D and cartoons were created in Pymol. (**d**) Partial LSD1 amino acid sequence indicating potential PKC-θ phosphorylation motifs near the NLS region (described in^45^). Red amino acids = NLS region; blue amino acids = potential PKC-θ phosphorylation sites. (**e**) LSD1-s111p TNFI in indicated cell lines (n > 30 individual cells). Representative images for each dataset are shown. (**f**) Graph indicates LSD1-s111p TNFI after treatment with vehicle alone, BIM or C27 as determined by immunofluorescence microscopy (n > 20 individual cells). (**g**) LSD1 H3K4 demethylation assay was performed on nuclear extracts from indicated cells after treatment with BIM, C27, or vehicle alone. Graph depicts percentage LSD1 demethylase activity relative to control (n = 2). (**h**) PCC for LSD1 and Snail after treatment with either BIM, C27, or vehicle alone (n ≥ 10 individual cells). −1 = inverse of colocalization; 0 = no colocalization; +1 = perfect colocalization. (**i**) LSD1-WT and LSD1-Mut plasmid construct sequences. Blue amino acid = mutation site. (**j**) Graphs indicate LSD1-s111p TNFI, vimentin TCFI, and Snail TNFI after incubation with LSD1-WT, LSD1-Mut, or vector only as determined by immunofluorescence microscopy (n = 40 individual cells). Scale bars = 10 µM. All data represents the mean ±SE. ns = p > 0.05; *P < 0.05; **P < 0.01; ***P < 0.001; ****P < 0.0001, Mann-Whitney test.

We have recently shown that protein kinase C-theta (PKC-θ) directly regulates inducible gene expression signatures during breast cancer EMT and in CSCs^38^, and our studies have shown that PKC-θ tethers to the chromatin template in complex with LSD1 to express inducible immunological response genes in T-cells^43^. We initially questioned whether LSD1 and PKC-θ co-exist during EMT and in dedifferentiated mesenchymal cells. Double staining and immunofluorescence microscopy showed that LSD1 and PKC-θ do not colocalise in epithelial MCF-7 cells. However, upon EMT, LSD1 and PKC-θ display moderate colocalization in MCF-7/PMA + TGF-β cells (PCC = 0.278) and high colocalization in fully dedifferentiated mesenchymal MDA-MB-231 cells (PCC = 0.519) (Fig. 3a).

Given that PKC-θ and LSD1 colocalise during EMT and in the mesenchymal state, we explored whether PKC-θ directly phosphorylates LSD1 using an array-based *in vitro* kinase assay. Of 201 overlapping peptide constructs, 15 were positive for phosphorylation events (top 10 shown in Supplementary Fig. 4). Sequence analysis of these 15 peptides showed that three peptides including the top two hits, were localised to the nuclear localisation signal (NLS) region (highlighted in red; Supplementary Fig. 4b). The peptide with the strongest phosphorylation signal (peptide 1) was focused on serine-111 (highlighted in blue) near the NLS domain. Since this phosphorylation site is directly adjacent to the NLS, it is likely that serine-111 is accessible and amenable to phosphorylation, as functional NLS domains are externalised and allow interaction with nuclear import receptors. A model of the full length LSD1 protein was generated by Phyre2^44^ highlighting the accessibility of serine-111 and the close proximity of the highly-positively charged NLS domain (Fig. 3c). A serine/threonine-specific motif for PKC-θ-mediated phosphorylation has been identified^45^, so we also examined the relationship between phosphorylated peptides in the NLS region of LSD1 and identified PKC-θ motifs (Fig. 3d): the strongest peptide signals (peptides 1 and 2) contained three PKC-θ consensus phosphorylation motifs. Overall, these data suggest that nuclear PKC-θ directly phosphorylates LSD1 at multiple regions including the NLS region, most likely at serine-111.

We next investigated whether LSD1 serine-111 phosphorylation (LSD1-s111p) occurs *in vivo* during EMT. Immunofluorescence microscopy revealed that LSD1-s111p was entirely nuclear, and the LSD1-s111p TNFI significantly increased in MCF-7/PMA + TGF-β and MDA-MB-231 cells relative to epithelial MCF-7 cells (Fig. 3e). On inhibiting PKC-θ with either C27 or BIM, the TNFI of LSD1-s111p was significantly reduced in all cell types except MCF-7 cells with BIM (Fig. 3f). This strongly indicates that PKC-θ is critical for phosphorylation of LSD1 at serine-111 and that this is important for nuclear localization. Inhibiting PKC-θ also increased the H3K4 demethylase activity of LSD1 (Fig. 3g), indicating that active PKC-θ may prevent LSD1 from demethylating H3K4 and subsequently causing gene repression. Moreover, PKC-θ inhibition with either agent significantly reduced LSD1 and Snail colocalization in both cells types (Fig. 3h).

To test the functional importance of serine-111 phosphorylation in EMT and CSCs, two plasmids were constructed: one expressing wild-type LSD1 (LSD1-WT) and the other containing a mutation at serine-111 to alanine (LSD1-Mut) to prevent phosphorylation at this site (Fig. 3i). Transfection with LSD1-WT but not LSD1-Mut significantly increased LSD1-s111p, vimentin, and Snail expression in MCF-7 cells whereas transfection of MCF-7/PMA + TGF-β with the LSD1-Mut abrogated this induction (Fig. 3j). Collectively, PKC-θ can directly phosphorylate LSD1 at serine-111 to cause nuclear localization, which decreases the repressive activity of LSD1 during EMT and regulates the expression of the EMT markers vimentin and Snail.

### Co-binding of PKC-θ and LSD1 to the chromatin template promotes transcription during EMT

**Figure 4:**
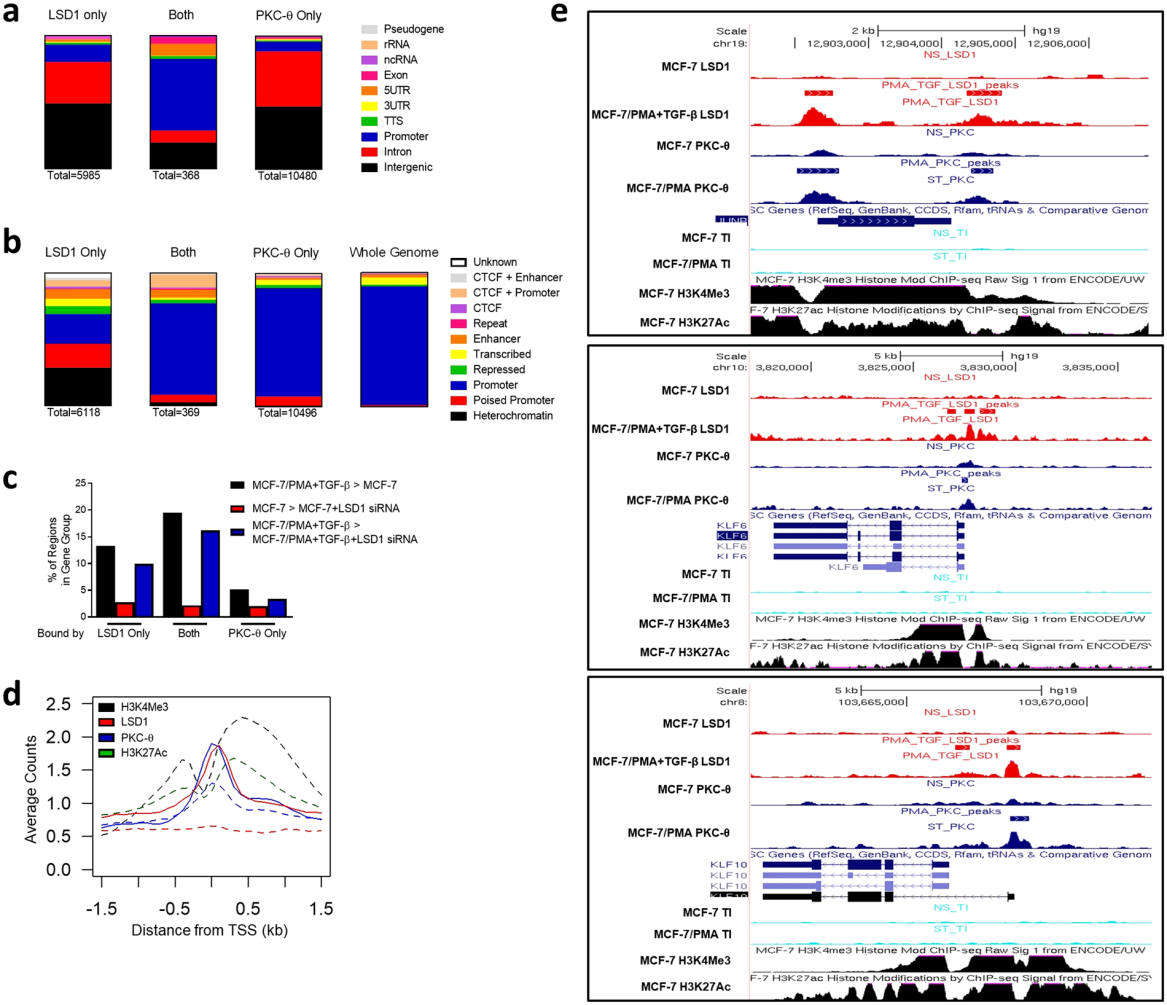
LSD1 and PKC-θ co-binding promotes transcription in mesenchymal cells. Diagram depicts proportion of peaks occupied by PKC-θ in MCF-7/PMA cells, LSD1 in MCF-7/PMA + TGF-β cells, and both PKC-θ and LSD1. LSD1 and PKC-θ ChIP-seq peaks and regions with both LSD1 and PKC-θ peaks were annotated to: (**a**) summarised chromatin environments of the nearest Ensembl transcript in different cell types. Peaks were classified as rRNA, 5’UTR, promoter (<1 kb from TSS), intergenic, intron, exon, pseudo, TTS, ncRNA or 3′UTR; and (**b**) summarised CTCF/chromatin states in MCF-7 cells. (**c**) Percentage of regions bound by LSD1 alone, both LSD1 and PKC-θ, or PKC-θ alone in the indicated gene groups where the number of regions is used as the denominator. (**d**) Average LSD1, PKC-θ, H3K4me3, and H3K27ac levels around the TSS of LSD1-sensitive genes. Reads are binned by 0.1 kb, ±1.5 kb around TSS. Dashed lines = MCF-7 samples; solid lines = MCF-7/PMA (PKC-θ) or MCF-7/PMA+ TGF-β (LSD1). (**e**) LSD1, PKC-θ, H3K4me3, H3K27ac, H3K9me3, and H3K27me3 ChIP-seq peaks at *JUNB*, *KLF6*, and *KLF10* in indicated cell types as shown in the UCSC Genome Browser.

Since PKC-θ and LSD1 can both act as chromatin-anchored proteins, we were interested in determining whether they co-bound to chromatin in the mesenchymal state. Using our previously published MCF-7 PKC-θ ChIP-seq data (MCF-7/PMA cells)^38^, we determined that of the 6487 regions bound by LSD1 (MCF-7/PMA + TGF-β cells), 368 were enriched for PKC-θ (PCC = 0.04). However, when the regions were restricted to those within 1 kb of a TSS, the PCC increased to 0.14, and, when further restricted to those near a TSS of an inducible LSD1-sensitive gene, the PCC increased to 0.64. Furthermore, 63% of regions bound by both LSD1 and PKC-θ were annotated as promoters/5′ UTR (Fig. 4a) and 84% had promoter marks in MCF-7 cells (Fig. 4b).

We next annotated each region bound by both LSD1 and PKC-θ to the nearest gene and overlaid this with MCF-7 and MCF-7/PMA + TGF-β LSD1 siRNA microarray data. 19% of the co-bound regions were annotated to genes that were inducible whilst 16% were annotated to genes whose expression was reduced in the presence of LSD1 siRNA in MCF-7/PMA + TGF-β cells. The percentages for regions bound by LSD1 only were significantly lower (p = 0.001 and 0.0001, respectively) (Fig. 4c). GO enrichment revealed that co-bound genes were enriched for several pathways and processes including: hippo, p53, TGF-β, and MAPK, as well as those for cell metabolism, gene regulation, and chromatin assembly.

Next, we profiled LSD1, PKC-θ, H3K4me3, and H3K27ac around the TSSs of LSD1-sensitive genes (Supplementary Fig. 5). LSD1 and PKC-θ binding increased at the TSSs between H3K4me3/H3K27ac (Fig. 4d, Supplementary Fig. 5) after EMT. Moreover, their binding appeared to target H3K4me3-high promoters, suggesting that they might assist in transcribing these genes. Of the genes directly tethered by LSD1 and PKC-θ, several were EMT-TFs including *JUNB*, *KLF10*, and *KLF6* in regions flanked by H3K4me3 and H3K27ac (Fig. 4e). Taken together, these data suggest that although PKC-θ and LSD1 predominantly occupy separate genomic compartments, when they co-bind this occurs primarily at promoters and near a higher proportion of genes that are induced and are LSD1-sensitive. PKC-θ appears to promote LSD1’s role in transcriptional regulation.

### LSD1 is involved in breast cancer growth and is enriched in chemoresistant cells

**Figure 5:**
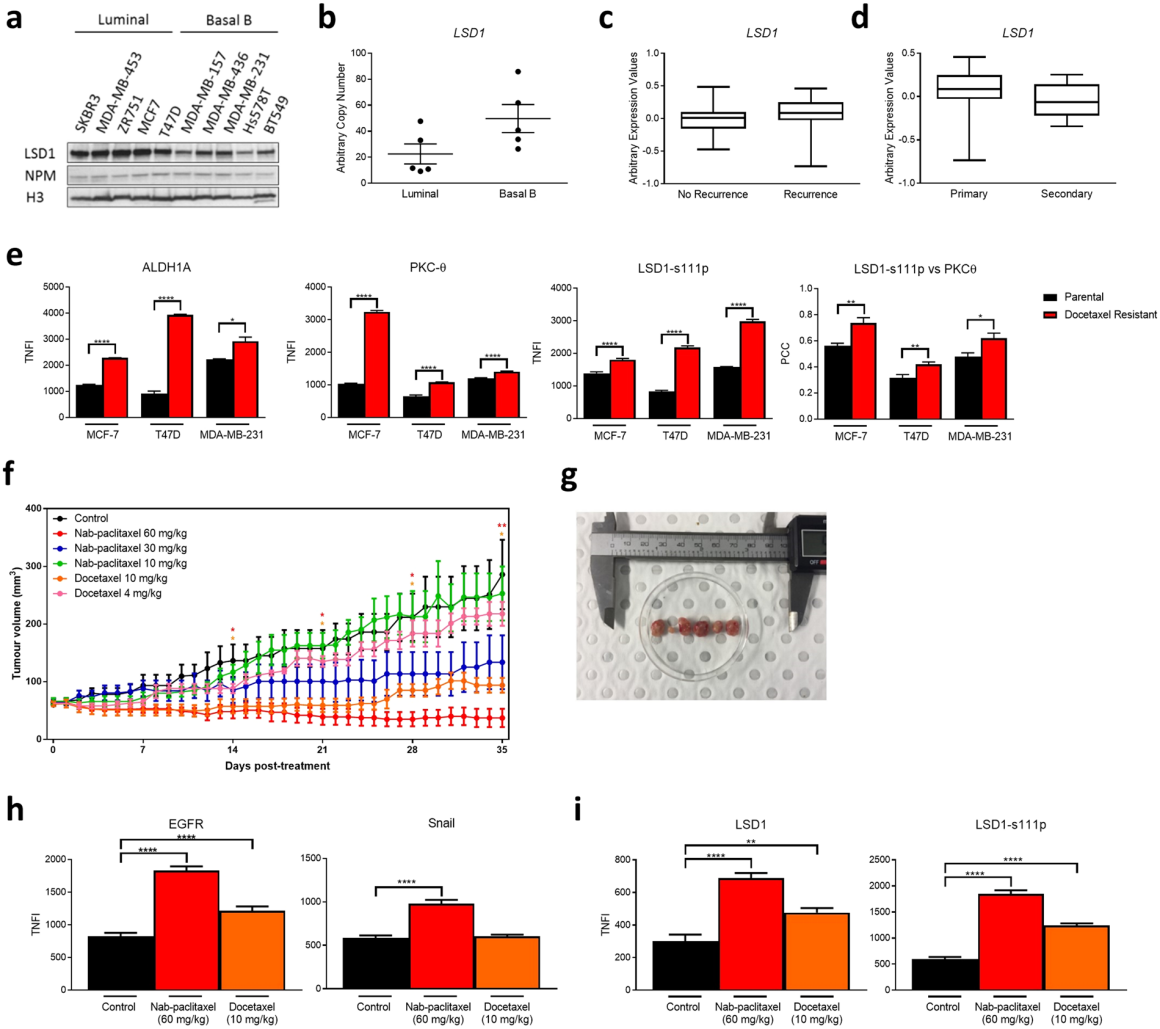
LSD1 is involved in breast cancer growth and is enriched in chemoresistant cells. (**a**) Western blots of indicated breast cancer cell line total cell extracts probed for LSD1. NPM and histone H3 were loading controls. Copped images are from samples and antibodies processed and run on the same gel. Full-length blot is shown in Supplementary Fig. 6. (**b**) *LSD1* mRNA levels as measured by qPCR in breast cancer cell lines characterised as ER positive and ER negative. Data are expressed as arbitrary copy numbers normalised to *PPIA* (n = 2). LSD1 expression in: (**c**) local recurrent and non-recurrent breast carcinomas from GEO dataset GSE4913 (n ≥ 19); (**d**) primary and secondary locally recurrent breast carcinomas from GEO dataset GSE4913 (n ≥ 9); (**e**) Immunofluorescence microscopy was performed on matched parental and docetaxel-resistant MCF-7, T47D and MDA-MB-231 cells fixed and probed with anti-ALDH1A1, anti-PKC-θ and anti-LSD1-s111p. Graphs indicate ALDH1A, PKC- θ and LSD1-s111p TNFI and PKC and LSD1-s111p PCC (n = 20 individual cells). −1 = inverse of colocalization; 0 = no colocalization; +1 = perfect colocalization. (**f**) Tumour growth (mm^3^) up to 5 weeks post-treatment with indicated treatments (n = 5). Coloured asterisk (*) denotes significance at that time point. (**g**) Tumours sizes 5 weeks post-treatment from left to right: control, nab-paclitaxel 60 mg/kg, nab-paclitaxel 30 mg/kg, nab-paclitaxel 10 mg/kg, docetaxel 10 mg/kg, and docetaxel 4 mg/kg. Tumours were excised and digested into single cell suspension and then subjected to immunofluorescence microscopy where TNFI was determined for control, nab-paclitaxel 60 mg/kg, and docetaxel 10 mg/kg treated tumours after probing with: (**h**) anti-EGFR, anti-Snail; or (**i**) anti-LSD1, or anti-LSD1-s111p (n > 10). All data represents the mean ± SE. *P < 0.05; **P < 0.01; ***P < 0.001; ****P < 0.0001, Mann-Whitney test.

We were interested in understanding the impact of LSD1 expression on tumourigenicity and treatment resistance given: (i) the role that it plays in EMT and CSC regulation; and (ii) a CSC-phenotype is linked with resistance to chemotherapy and endocrine therapy^6,46^. LSD1 expression was first analyzed in a panel of luminal (SKBR3, MDA-MB-453, ZR751, MCF-7 and T47D) and basal B (MDA-MB-157, MDA-MB-436, MDA-MB-231, Hs578T and BT549) breast cancer cell lines^47^. LSD1 expression was higher in the less aggressive luminal cell lines than the aggressive basal B cell lines (Fig. 5a; full image shown in Supplementary Fig. 6). There was higher *LSD1* expression in basal b cell lines (Fig. 5b) which are ER^−^ and associated with poorer patient prognosis in clinical studies^48^. However, the repressive H3K4 demethylase activity of LSD1 is higher in luminal MCF-7 cells then basal B MDA-MB-231 cells (Fig. 1e). We speculate that while LSD1 levels are higher in luminal cancer cell lines it may have a more activatory role in aggressive basal B cancer cell lines. Using publicly available data, we found that *LSD1* expression was higher in the breast cancers of patients who recurred after treatment than those who did not (GSE4913; Fig. 5c). Of the patients who recurred after treatment, *LSD1* expression was higher in the primary carcinoma than the recurrent carcinoma (GSE4913; Fig. 5d). We further analyzed LSD1 levels in MCF-7, T47D and MDA-MB-231 cells that had developed resistance to docetaxel by stepwise exposure to the taxane (as described in^49^). Interestingly, we found that these cells were enriched in the stem-like marker, aldehyde dehydrogenase 1 family member A1 (ALDH1A1) which is associated with both poor cancer prognosis and a CSC phenotype^50^, as well as PKC-θ and LSD1-s111p and moreover, there was increased colocalization between PKC-θ and LSD1-s111p (Fig. 5e). Collectively, these data suggest that LSD1 is involved in enriched in chemotherapy-resistance cells whereby it may facilitate tumour progression and recurrence.

This prompted us to examine the involvement of LSD1 in the growth of cancer xenografts *in vivo*. MDA-MB-231 cells were transplanted subcutaneously into the mammary fat pads of balb/c nude mice and treated with intraperitoneal (IP) injections of: (1) vehicle control only; (2) nab-paclitaxel (60, 30, or 10 mg/kg); or (3) docetaxel (10 or 4 mg/kg) (details in Supplementary Fig. 7a). The tumour volume decreased in a dose-dependent manner after treatments with both nab-paclitaxel and docetaxel with significant decreases in tumour volume observed after two weeks treatment in mice treated with the highest dosages (Fig. 5f,g). The surviving tumour cells were profiled for their expression of Snail and epidermal growth factor receptor (EGFR), which has been linked with tumour metastasis and treatment resistance^51^. On immunofluorescence staining, the surviving nab-paclitaxel-resistant tumour cells showed a significant increase in Snail and EGFR expression and docetaxel-resistant tumour cells showed a significant increase in Snail (Fig. 5h), consistent with a more aggressive phenotype. Further, after both treatments, there was a significant increase in LSD1 and LSD1-s111p expression (Fig. 5i), thus confirming that LSD1 is enriched in chemoresistant cells.

### LSD1 inhibition ***in vivo*** suppresses tumour growth, chemotherapy-induced EMT, and CAFs

We next examined the effect of nab-paclitaxel and phenelzine – a well-characterised, LSD1 and monoamine oxidase inhibitor^52^ – alone or in combination on tumour growth and phenotype *in vivo*. Again, balb/c nude mice were injected subcutaneously into the mammary fat pads with MDA-MB-231 cells and treated by IP injections with: (1) vehicle only control; (2) nab-paclitaxel (30 mg/kg); (3) phenelzine (40 mg/kg); and nab-paclitaxel + phenelzine (previously indicated concentrations) (details in Supplementary Fig. 7b). Five weeks post-treatment there was a significant reduction in nab-paclitaxel alone treated tumour volume and this was potentiated when administered in combination with phenelzine treated mice (data not shown). We further validated these findings in the MDA-MB-231 balb/c nude mice model by treating with a combination of docetaxel (4 mg/kg) and pargyline, (100 or 200 mg/kg) another LSD1 inhibitor. Similarly, we found a reduction in tumour volume as early as one week post-treatment with both high and low dosages of LSD1 inhibitor (Supplementary Fig. 8).

**Figure 6:**
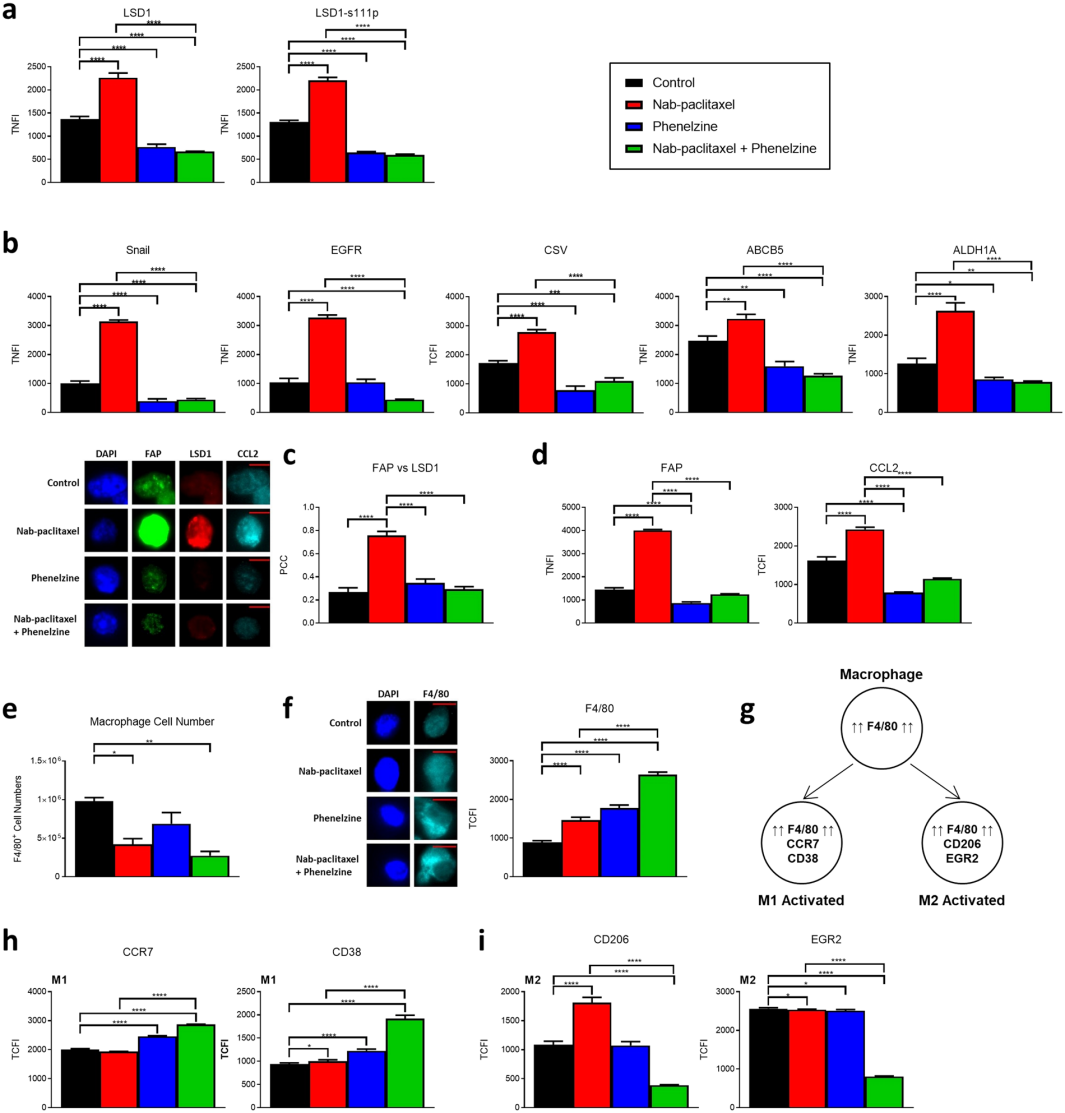
LSD1 inhibition suppresses chemotherapy-induced EMT, CAFs and promotes a M1 macrophage infiltration. Balb/c nude mice were treated with either vector only, nab-paclitaxel 30 mg/kg, phenelzine 40 mg/kg, or nab-paclitaxel 30 mg/kg + phenelzine 40 mg/kg. Tumours were excised, cut in half, digested with collagenase IV into single cell suspensions and immunofluorescence microscopy was performed on pooled samples and TNFI or TCFI was determined after probing with: (**a**) anti-LSD1, anti-LSD1-s111p; or (**b**) anti-Snail, anti-EGFR, anti-CSV, anti-ABCB5, or anti-ALDH1A1 (n ≥ 15 individual cells from 5 pooled). (**c**) PCC was determined for FAP and LSD1 (n = 20 individual cells from 5 pooled). −1 = inverse of colocalization; 0 = no colocalization; +1 = perfect colocalization. (**d**) Graphs indicate FAP TNFI and CCL2 TCFI (n = 20 individual cells from 5 pooled). Representative image is shown for each data set. Scale bars = 10 µM. (**e**) Graph represents the total number of F4/80^+^ cells in individual tumours for each group as determined by flow cytometry (n ≥ 4). (**f**) TCFI of F4/80 (n = 60 individual cells from 5 pooled). Representative image is shown for each data set. Scale bars = 20 µM. (**g**) All cells were probed with anti-F4/80 and either anti-CCR7, anti-CD38, anti- CD206 or anti-EGR2. Cells that were determined to be F4/80-positive were then analyzed for their expression of M1- or M2-activated macrophage markers. TCFI was then determined for (**h**) CCR7, CD38; (**i**) CD206 and EGR2 (n ≥ 40 individual cells from 5 pooled). Black = control; red = nab-paclitaxel; blue = phenelzine; green = nab-paclitaxel + phenelzine. All data represents the mean ± SE. *P < 0.05; **P < 0.01; ***P < 0.001; ****P < 0.0001, Mann-Whitney test.

We next examined the expression of LSD1 and LSD1-s111p in the nab-paclitaxel and phenelzine alone and combination treated tumours. LSD1 and LSD1-s111p TNFI significantly increased in surviving nab-paclitaxel treated tumour cells and decreased significantly in tumour cells surviving phenelzine alone or combination treated tumour cells (Fig. 6a). Following this, we characterised the molecular phenotype of these tumours by examining the fluorescent intensity of several EMT, stem-like, and resistance markers, namely Snail, EGFR, cell-surface vimentin (CSV), ALDH1A and ATP-binding cassette sub-family B member 5 (ABCB5). CSV, ALDH1A and ABCB5 are all key markers of metastatic breast cancer (MBC) circulating tumour cells (CTCs). CTCs expressing CSV have been linked with poor prognosis in a variety of cancers^53,54^ whilst ALDH1A expression is linked with metastasis in solid tumours and early recurrence^55,56^. Similarly, ABCB5 has been linked with poor prognosis and multi-drug resistance^57,58^. Our data demonstrates that each of these markers increased after treatment with nab-paclitaxel alone but decreased after treatment with phenelzine or phenelzine in combination with nab-paclitaxel (Fig. 6b). Our data suggests that LSD1 and LSD1-s111p are upregulated in chemoresistant tumour cells and moreover, these cells appear to have undergone a chemotherapy-induced EMT and display an upregulation of mesenchymal and stem-like resistance markers. Targeting LSD1 in combination therapy blockades this *in vivo* chemotherapy-induced EMT and suppresses tumour growth.

Given the documented role of tumour stroma and in-particular CAFs in cancer progression, we were interested to see if inhibiting LSD1 affects CAFs *in vivo*. To investigate this further protein levels of the CAF markers, FAP and CCL2 as well as LSD1 were analyzed by immunofluorescence microscopy. While FAP is usually described as a soluble or membrane-bound protein, high-resolution microscopy suggested a potential nuclear role. FAP and LSD1 weakly colocalised in control samples but colocalization increased significantly in nab-paclitaxel alone treated samples and decreased significantly after treatment with phenelzine alone or combination treatments (Fig. 6c). There was also a significant increase in total fluorescence intensity of FAP and CCL2 after nab-paclitaxel monotherapy that reduced significantly after LSD1 inhibition (Fig. 6d). These data indicate that CAFs increase in the tumour microenvironment after mono-chemotherapy but are reduced after LSD1 inhibition alone or in combination with chemotherapy.

Given that LSD1 has recently been implicated in the regulation of monocyte-to-macrophage differentiation^59^ and the lack of adaptive immunity in balb/c nude mice, we explored whether LSD1 may be modulating innate immunity through macrophage regulation. We found an overall decrease of F4/80^+^ cells by flow cytometry after all treatments indicating a reduction in macrophages (Fig. 6e). However, immunofluorescence microscopy indicated that F4/80 TCFI increased significantly in combination treated samples (Fig. 6f). As these data indicate an infiltration of macrophages we were interested in determining if they were classical (M1) or alternatively activated (M2) macrophages. Immunofluorescence microscopy was performed probing for the M1 markers CCR7 and CD38 and the M2 markers CD206 and EGR2. To confirm that these markers were being expressed by macrophages, cells were first screened for the presence of F4/80 staining and subsequently for co-expression of M1 or M2 markers (Fig. 6g). There was a significant increase in M1-associated CCR7 and CD38 after phenelzine alone and combination treatments (Fig. 6h). Conversely, there was a significant decrease in M2-associated CD206 and EGR2 after all treatments (Fig. 6i). Finally, haematoxylin and eosin staining of control, docetaxel-treated, and combined pargyline-docetaxel-treated tumours revealed an infiltrating front of immune cells at the periphery of the carcinomas after combination treatment that was less apparent in chemotherapy alone treated or control samples (Supplementary Fig. 9).

Taken together, these data demonstrate that LSD1 induces upregulation of a mesenchymal, stem-like, and resistance signature in cancer cells that survive chemotherapy. Combination treatment with a LSD1 inhibitor is advantageous as both the bulk tumour cell population and the mesenchymal, stem-like, resistance signature are significantly abrogated along with a reduction in CAF signature and increased M1 macrophage and innate immune cell infiltration potentially supporting the role of LSD1 in reducing recurrence.

### LSD1 is upregulated in CTCs isolated from MBC patient liquid biopsies and is associated with a mesenchymal- and CSC-like phenotype

**Figure 7:**
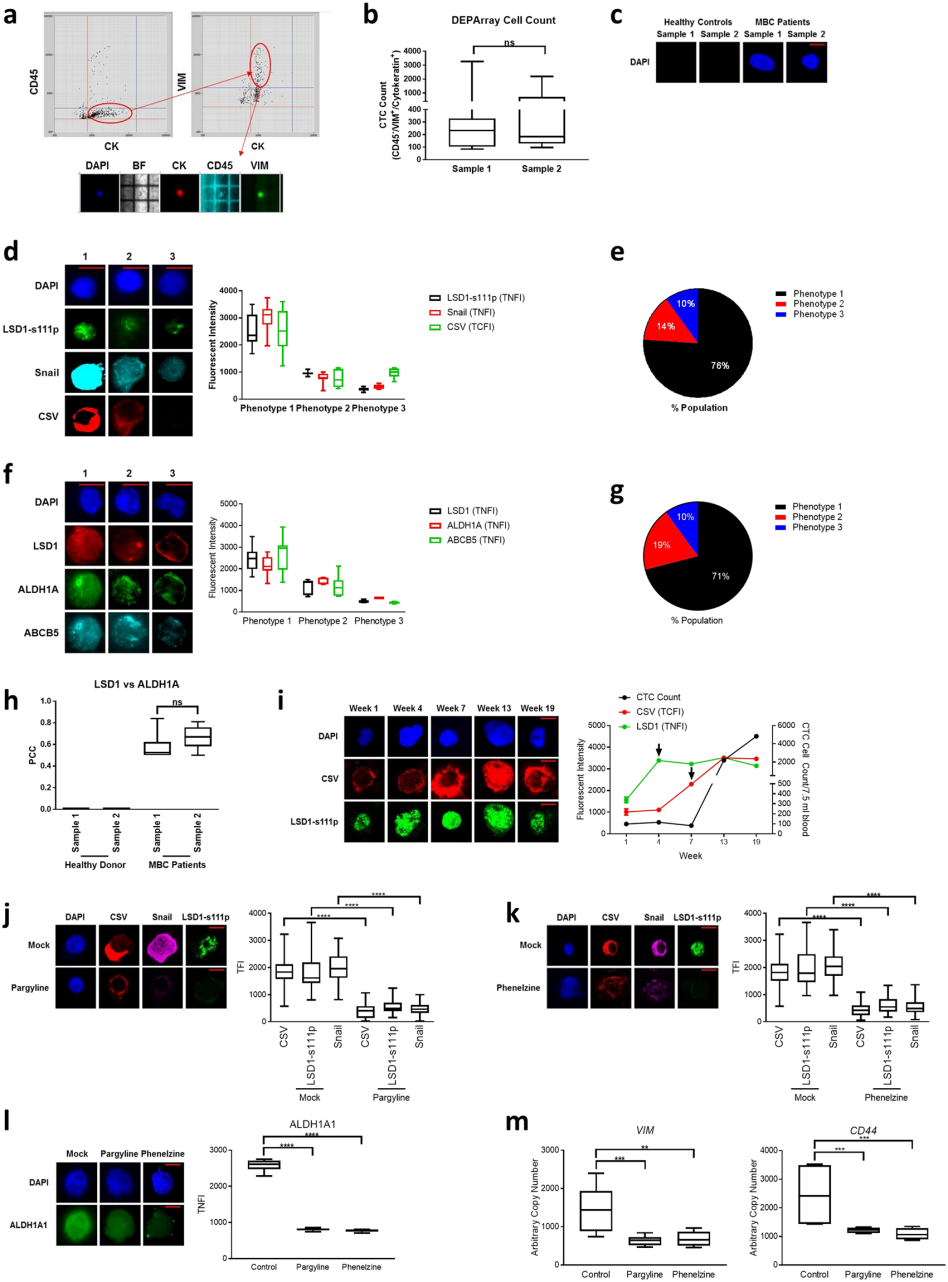
LSD1 correlates with MBC CTC mesenchymal- and CSC-like characteristics. (**a**) CTCs were isolated from MBC patient liquid biopsies through double expression of cytokeratin (CK) and vimentin (VIM) and absence of CD45 expression on the DEPArray. Representative image from DEPArray and workflow shown. (**b**) CD45^−^/VIM^+^/CK^+^ CTC cell counts were determined in two samples collected six weeks apart (sample 1 and sample 2, respectively) (n = 10). (**c**) Immunofluorescence microscopy was performed on all fixed CTCs collected from 10 MBC patients at each time point and compared to normal donors. Representative images showing DAPI staining in CTCs is shown (n = 5 healthy donors and 10 MBC patients). (**d**) CTCs were separated based on LSD1-s111p TNFI relative to negative controls, primary and secondary antibody controls as well as healthy donor expression into phenotypes 1, 2 and 3 (high, moderate and low expression, respectively) and Snail TNFI and CSV TCFI were determined (n = 10). Representative images are shown for each dataset. (**e**) Average percentage of phenotype 1, 2 and 3 cells (n = 10). (**f**) CTCs were separated based on LSD1 TNFI relative to negative controls, primary and secondary antibody controls as well as healthy donor expression into phenotypes 1, 2 and 3 (high, moderate and low expression, respectively) and ALDH1A TNFI and ABCB5 TNFI were determined (n = 10). Representative image is shown for each dataset. (**g**) Average percentage of phenotype 1, 2 and 3 cells (n = 10). (**h**) Graph depicts the PCC for LSD1 and ALDH1A (n = 10). −1 = inverse of colocalization; 0 = no colocalization; +1 = perfect colocalization. (**i**) Graph depicts LSD1-s111p TNFI, CSV TCFI and CD45^−^/VIM^+^/CK^+^ CTC cell count in five samples collected at indicated time points. Representative images are shown for each time point (n = 1 patient). Isolated CTCs were treated with either vehicle alone, pargyline or phenelzine and then immunofluorescence microscopy or qPCR was performed. Total florescence intensity of CSV and LSD1-s111p and Snail after treatment with (**j**) pargyline; or (**k**) phenelzine (n > 75 cells from 10 patients). (**l**) ALDH1 TNFI after treatment with pargyline or phenelzine (n = 20 cells from 10 patients). (**m**) *VIM* and *CD44* mRNA transcript levels after treatment with pargyline or phenelzine (n = 10 pooled). Scale bars = 10 μM. ns = p > 0.05; *P < 0.05; **P < 0.01; ***P < 0.001; ****P < 0.0001, Mann-Whitney test.

Given that our results thus far strongly suggest that LSD1 is involved in a multitude of breast cancer programs, we investigated the role of LSD1 in promoting mesenchymal and CSC-like CTCs from patients with Stage IV MBC. CTCs are a crucial component of the metastatic cascade, tumour dissemination, progression and tumour recurrence/relapse. The study recruited 10 patients classified as having stage IV MBC who at baseline were ER^+^/PR^+^/HER2^−^ and had received any form of systemic therapy. CTCs from liquid biopsies were identified using the DEPArray based on high expression levels of vimentin, cytokeratin (CK) and the absence CD45 expression (Fig. 7a). CD45^−^/VIM^+^/CK^+^ CTCs were counted and then isolated from all patients at two time points, six weeks apart during standard of care (sample 1 and sample 2, respectively) (Fig. 7b). All MBC patient liquid biopsies contained CTCs, in contrast, no CTCs were detected from healthy donors (Fig. 7c).

We characterised isolated CTCs to determine if they expressed markers indicating a mesenchymal phenotype. High resolution fluorescent microscopy demonstrated that the MBC CTCs could be separated into three distinct groups based on their expression of LSD1-s111p or LSD1. Examining the co-expression of LSD1-s111p, Snail and CSV revealed that cells expressing high levels of LSD1-s111p (TNFI >1100; denoted phenotype 1) also expressed high levels of Snail and CSV. Whilst phenotypes 2 and 3 which expressed moderate (TNFI 500–1100) and low (TNFI <500) levels of LSD1-s111p, had moderate and low levels of EMT markers, Snail and CSV, respectively, demonstrating that LSD1-s111p levels are proportional to the mesenchymal status of CTCs (Fig. 7d). Interestingly, phenotype 1 cells comprised the bulk of the MBC CTCs (76%), while 14% where phenotype 2 and 10% were phenotype 3 (Fig. 7e). To further investigate the co-expression signature of these CTCs we examined a panel of stem like-resistant biomarkers including ALDH1A and ABCB5 in the context of LSD1 expression^50,60^. Similarly, we determined that within this panel, phenotype 1 comprised 71% of the CTCs and co-expressed high levels of LSD1 (TNFI >1500) and the stem-like markers, ALDH1A and ABCB5. Phenotype 2 (19%) and phenotype 3 (10%) CTCs expressed moderate (TNFI 600–1500) and low (TNFI <600) levels of LSD1 correlating ALDH1A and ABCB5 expression (Fig. 7f,g). Moreover, LSD1 strongly colocalised with ALDH1A (Fig. 7h).

Next, we investigated whether LSD1-s111p and CSV along with CTC count was altered with MBC disease progression. Thus, we tracked the fluorescent intensity of these markers and CTC count in one patient at numerous time points. Standard of care monitoring (CT scans (RECIST 1.1), blood work, clinical symptoms/judgement) indicated that progression of metastatic disease and increasing disease burden was accompanied by an increase in CTC count in this patient. Intriguingly, there was a spike in the co-expression of both LSD1-s111p and CSV that preceded the substantial increase in CTC count (indicated by arrows) (Fig. 7i).

To further investigate the role of LSD1 in CTC regulation, we treated isolated MBC patient CTCs with vehicle alone, pargyline or phenelzine and monitored the expression of mesenchymal protein biomarkers. Inhibition of LSD1 significantly reduced the fluorescent intensity of CSV, Snail and LSD1-s111p after both pargyline and phenelzine treatment (Fig. 7j and k, respectively). Additionally, treatment with either pargyline or phenelzine significantly reduced the TNFI of the CSC marker, ALDH1A (Fig. 7l). Similarly, we found significant reductions in *VIM* and *CD44* mRNA transcript levels by qPCR (Fig. 7m). Overall, these results demonstrate that LSD1 has a role in promoting both the mesenchymal and CSC signature of MBC CTCs. Abrogation of LSD1 mediates a reduction of CSC burden whilst reducing the mesenchymal status suggesting that LSD1 maybe be a critical target in lessening the CSC and disease burden in MBC patients.

## Discussion

In response to inflammatory signal-mediated EMT, *LSD1* expression was induced, subsequently peaked, and then returned to baseline upon complete dedifferentiation. Although *LSD1* mRNA levels reverted to baseline, LSD1 protein persisted and colocalised with the key EMT-TF Snail during EMT and regulated the expression of several EMT markers including Snail, vimentin, and E-cadherin. These findings are consistent with previous reports that LSD1 regulates transcription of EMT pathways in breast cancer^22,29^. However, the molecular mechanisms through which LSD1 regulates EMT and other aspects of cancer progression is only beginning to be elucidated.

In the present study, we have demonstrated that LSD1 regulated widespread genomic changes during EMT that result in active induction of CSC phenotype mediated by increased expression of multiple EMT-related genes. Of the genes that were LSD1-sensitive, the majority were induced rather than repressed during EMT. This is consistent with the decrease in repressive LSD1-mediated H3K4 demethylase activity we observed in mesenchymal-like cells. Importantly, of the induced LSD1-sensitive genes, many were EMT related including the regulation of cell migration, motility, and differentiation^2^ and EMT pathways such as TGF-β and PI3K-Akt signaling^61^.

We found that upon induction of EMT, LSD1 occupancy shifted from preferentially binding at intergenic and intronic regions to binding a larger proportion of promoter regions. Moreover, LSD1 binding increased at regions annotated as active promoters and reduced in those annotated as repressed chromatin in MCF-7 cells^40^. These findings differ from those in embryonic stem cells (ESC), in which LSD1 largely lies on the enhancers of actively transcribed and bivalent genes^62^; indicating that LSD1 binding is likely to be cell type specific. Although we found that LSD1 induced a large number of genes, around 86% of these were not tethered by LSD1. LSD1 did, however, directly regulate the expression of several EMT-TFs including *JUNB*, *KLF10*, and *KLF6*, probably indirectly through the action of directly targeted LSD1-sensitive TFs.

This study demonstrates for the first time that PKC-θ interacts with LSD1 during breast cancer EMT, with LSD1 and PKC-θ colocalizing in mesenchymal-like cells but not in epithelial cells. At the chromatin level, PKC-θ and LSD1 bound separate genomic regions in MCF-7 cells, however, in cells that have undergone EMT both proteins predominantly co-occupied promoter regions of genes induced during EMT in a LSD1-dependent manner. Indeed, our data indicated that LSD1 and PKC-θ co-binding occurred between H3K4me3 and H3K27ac flanked regions, targeted H3K4me3 rich promoters, and occurred predominantly at induced LSD1-sensitive genes. Previously, PKC-β has been shown to colocalise with LSD1 and the androgen receptor (AR) at target gene promoters, where PKC-β mediates H3T6 phosphorylation to facilitate gene expression^63^. We propose that a similar mechanism occurs during breast cancer EMT, with PKC-θ occupying select promoter regions with LSD1 and acting to enhance LSD1-mediated gene induction in response to inflammatory signal-induced EMT.

We exploited peptide microarray kinase profiling to show that PKC-θ directly phosphorylates LSD1 at multiple sites. Several of these regions were within the NLS of LSD1, especially serine-111, whose phosphorylation was (i) dependent on PKC-θ activity and (ii) increased in the nucleus to facilitate vimentin and Snail induction in mesenchymal-like cells. Moreover, PKC-θ inhibition increased repressive LSD1 H3K4 demethylase activity, indicating that PKC-θ normally reduces LSD1-mediated repression. In *in vivo* mice models, PKC-α has been shown to phosphorylate mouse LSD1 at serine-112 (analogous to serine-111 in humans) in a cardiac rhythm-dependent manner and in breast cancer EMT and metastasis to enable E-box-mediated transcription^64,65^. Here we show for the first time that PKC-θ directly phosphorylates LSD1 and facilitates breast cancer EMT. Recent work has shown that the histone acetyltransferase, MOF, acetylates LSD1 at lysine residues 432, 433 and 436 in epithelial cells thereby displacing LSD1-chromatin interactions and suppressing EMT^66^. We speculate that a reduction in MOF levels precedes EMT, followed by phosphorylation of LSD1 at serine-111 by PKC-θ thus facilitating EMT by allowing LSD1 translocation to the nucleus, chromatin docking and subsequent demethylation of target genes.

We found that *in vivo*, chemotherapy was sufficient to reduce the primary tumour bulk, however, our data strongly suggests that LSD1 participates in the malignant phenotype and that its inhibition in combination with chemotherapy further suppresses tumour growth. LSD1-s111p was enriched in chemoresistant mesenchymal tumour cells after cytotoxic monotherapy, where it may play a role in chemotherapeutic resistance as suggested by our data demonstrating an increase in EGFR, ALDH1A and ABCB5 in these cells. Moreover, targeting LSD1 significantly reduced features of EMT and a stem-like resistance signature that were induced after chemotherapy, as well as the expression of both LSD1 and its phosphorylated form. These results are consistent with findings that LSD1 modifies the chemosensitivity of mouse hepatocytes undergoing TGF-β-induced EMT^23^.

Our *in vitro* data indicated that LSD1 selectively induces CSC genes whilst repressing NCSC subsets to promote cancer progression. Aside from regulating transcription in these cells, our results suggest that LSD1 may also regulate CSCs indirectly through the action of CAFs. LSD1 colocalised with the activated CAF marker, FAP, after nab-paclitaxel monotherapy, and targeting LSD1 in a combination therapy reduced CAF markers that were induced by the chemotherapy alone. Given the CAFs are implicated in regulating CSC self-renewal through paracrine mechanisms via increased secretion of cytokine CCL2^35^, LSD1 may also regulate breast CSC self-renewal by modulating the tumour microenvironment. Moreover, our data strongly suggests that LSD1 represses the innate immune response by regulating macrophage infiltration to the primary tumour site. Recent work has suggested that LSD1 may be involved in repressing monocyte-to-macrophage differentiation after THP-1 treatment potentially via H3K4 methylation at target gene promoters^59^. It is possible that a similar mechanism is in play here.

Data acquired from isolated CTCs from liquid biopsies of 10 MBC patients revealed that LSD1 is enriched in CTCs with mesenchymal, stem-like phenotypes. LSD1 expression increases during breast cancer progression and precedes detectable changes in CTC load, suggesting that LSD1 expression may be a predictive marker of disease prognosis. We found that LSD1 colocalises with stem-like markers in CTCs and pharmacological inhibition of LSD1 reduces the CSC- and mesenchymal-like phenotype of these cells. This suggests that not only may LSD1 be a predictive biomarker, it may regulate the metastatic properties of CTCs thereby regulating tumour progression. However, one limitation of our study is the small numbers of CTCs that can be recovered with current technologies, which in turn limits the scope and breadth of detailed epigenetic analysis at the individual patient level. As these CTC recovery and epigenetic based technologies improve for primary cell work this shortfall of cell numbers can be addressed allowing for more detailed epigenetic analysis. Additionally, whilst outside the purview of this study, it is of importance to investigate how multiple variables including cancer stage (Stage I to IV), hormonal status and therapy received each influence the expression of LSD1 in CTCs from patients with MBC.

We therefore propose a model in which LSD1 expression is transiently increased due to inflammatory signals induced at the primary tumour site. A global increase in LSD1 levels (Fig. 1b) coincides with a shift in LSD1 binding to promoter regions (Fig. 2c,d) and subsequently, LSD1 initiates genomic programs that promote EMT (Fig. 2b). Moreover, LSD1 selectively induces gene expression in a subpopulation of CSCs (Fig. 2i) and due to the chemoresistant nature of such CSCs, LSD1 is overrepresented after chemotherapy (Fig. 5e,i,a). LSD1 promotes other aspects of tumour progression by increasing CAFs (Fig. 6d), and repressing a M1 macrophage immune infiltrate at the primary tumour site (Fig. 6h). Additionally, LSD1 promotes the expression of mesenchymal characteristics of CTCs that have shed from the primary tumour into the circulatory system (Fig. 7j–m). However, whilst our *in vitro* data suggests that an increase in LSD1 occurs in response to increased inflammatory signalling (Fig. 1a,b), further experiments will be needed to validate that this occurs at the primary tumour site. Such studies should examine the correlation between key pro-inflammatory cytokines such as IL-1β, IL-6, and TNF-α and LSD1 levels.

Here we explored the role of LSD1 in breast cancer EMT, CSCs, and therapeutic resistance. We showed that LSD1 is reversibly induced during EMT where it regulates inducible EMT-related gene expression programs, primarily by PKC-θ-mediated phosphorylation of LSD1 at serine-111. *In vivo*, LSD1 inhibition in combination with chemotherapy represses tumour growth by inhibiting the mesenchymal, CAF and CSC phenotypes that were induced by chemotherapy alone. Additionally, combined inhibition increased innate immunity by regulating macrophage infiltration. Overall, our findings suggest that altering the LSD1 landscape can prime cancers, the stromal microenvironment, and immune cells for optimal chemotherapeutic action.

Establishing exactly how LSD1 regulates cell fate in the face of chemotherapy will potentially facilitate the development of epigenetic therapies that specifically target these mechanisms. Epigenetic therapies are prime candidates for adjuvant treatment with either chemotherapy or immunotherapy. Thus, understanding how LSD1 and LSD1-s111p differentially regulate such processes is paramount in selecting and designing effective treatment regimes. Current clinical trials in this space utilizing epigenetic drugs in combination therapy are showing promise in treating metastatic cancers^67^. In this context and in light of our findings, a phase 1 clinical trial would be most efficacious by combining a selective LSD1 inhibitor or PKC-θ inhibitor – to prevent serine-111 phosphorylation – with standard of care for patients with MBC. Such combination therapeutic strategy would likely improve patient survival by targeting the specific form of LSD1 modulating the tumour microenvironment to impede not only tumour burden but recurrence resulting in a favorable anti-tumour environment.

## Methods

### Cell culture

All breast cancer cell lines used were sourced from ATCC, except the docetaxel resistant lines which were kind gift of Dr Sikic (Standford University). Cell lines were maintained and cultured in DMEM (Invitrogen) supplemented with 10% FBS, 2 mM L-glutamine, and 1% PSN. MCF-7 cells were stimulated with 1.29 ng/ml phorbol 12-myristate 13-acetate (PMA) (Sigma-Aldrich) and 5 ng/ml recombinant TGF-β1 (R&D Systems) for 60 hours^38^. For inhibitor experiments, 10 µM, 1 µM and 0.1 µM erlotinib (Roche), 3 mM pargyline (Sigma-Aldrich), 500 µM phenelzine (Sigma-Aldrich), 1 µM BIM (Calbiochem), and 1 µM C27 (SYNthesis Med Chem) were used. All inhibitors were pre-incubated for 24 hours before stimulating cells. Forward transfection reactions were performed with 40 nM human siRNA (sc-60970) and mock siRNA (sc-36869) (Santa Cruz Biotechnology) using Lipofectamine 2000 (Invitrogen). Vector transfections were performed with 15 µg of either vector only plasmid (pTracer-CMV/BSD, Thermo Scientific), LSD1-WT, or LSD1-Mut (Serine-111 to Alanine) using the NEON^TM^ Transfection System kit (MPK5000; Invitrogen).

### RNA extraction and quantitative real-time PCR

Total RNA was extracted from cells as previously described^68^. 1 μg of extracted RNA was subsequently treated with recombinant DNase I (Roche) and reverse-transcribed using the Maxima First Strand cDNA Synthesis Kit (Thermo Scientific) according to the manufacturer’s protocol. TaqMan quantitative real-time PCR was performed as previously described^68^. Human TaqMan probes utilised were: *LSD1* (Hs01002741_m1), *CDH1* (Hs00170423_m1), *VIM* (Hs00958111_m1), *SNAI1* (Hs00195591_m1), *CD44* (Hs01075861_m1), and *PPIA* (Hs99999904_m1).

### Immunofluorescence microscopy

Immunofluorescence microscopy was performed to determine the TNFI, TCFI and PCC as previously described^69,70^. PCC values were determined by the strength of the relationship between two fluorochrome signals. Primary antibodies were: anti-rabbit-LSD1 (05–939; Merck Millipore), anti-goat-SNAI1 (sc-10433; Santa Cruz), anti-mouse-E-cadherin (sc-21791; Santa Cruz), anti-mouse-vimentin (sc-6260; Santa Cruz), anti-rabbit-PKC-θ-T538p (ab63365; Abcam), anti-rabbit-LSD1-s111p (ABE1462; Merck Millipore), anti-rabbit-ALDH1A1 (ab52492; Abcam), anti-rabbit-EGFR (ab2430; Abcam), anti-mouse-CSV (H00007431-M08; Abnova), anti-goat-ABCB5 (ab77549; Abcam), anti-rabbit-FAP-α (ab28244; Abcam), anti-goat CCL2 (sc-1304; Santa Cruz), anti-goat-F4/80 (sc-26642; Santa Cruz), anti-goat-CCR7 (NB100–712; Novus Biologicals), anti-mouse-CD38 (102761; Biolegend), anti-mouse-CD206 (ab8918; Abcam), and anti-goat-EGR2 (sc-204050; Santa Cruz). Secondary antibodies used: anti-rabbit-Alexa Fluor 488 (A21206; Life Technologies) or anti-rabbit-Alexa Fluor 568 (A10042; Life Technologies), anti-mouse-Alexa Fluor 568 (A10042; Life Technologies), or anti-goat-Alexa Fluor 633 (A21082; Life Technologies).

### LSD1 activity assay

LSD1 H3K4 Demethylation Activity Assay (KA1525, Abnova) was performed on nuclear extracts from MCF-7 and MDA-MB-231 cells in duplicate wells according to the manufacturer’s protocol.

### Flow cytometry

Flow cytometry and the CSC gating strategy were performed as in^38^. Briefly, cells were stained with anti-CD44-APC (559942; BD Biosciences), anti-CD24-PE (555428; BD Biosciences) antibodies, and Hoechst 33258 (94403; Sigma-Aldrich). Flow cytometry acquisition was performed on single cell suspensions using BLSR II. Analysis was performed using FlowJo software.

### Western blotting

Western blot analysis of total cell extracts from breast cancer cell lines was performed using primary anti-rabbit-LSD1 (ab17721; Abcam), anti-histone H3 (4499; CST), and anti-NPM (3542 S; CST) and secondary HRP-conjugated-anti-rabbit (A0545; Sigma-Aldrich). Signals were detected by the Super Signal Chemiluminescent ECL-plus (Amersham) with film exposure.

### Microarray analysis

Microarray studies were performed on MCF-7 and MCF-7/PMA + TGF-β cells treated with either mock or LSD1 siRNA. Hugene 2.0 ST arrays were normalised using RMA in Affy Power Tools 1.16.1. Only probes annotated as ‘main’ were used. Probe sets were called differentially expressed if greater than log_2_ 0.5 different.

### ChIP and ChIP-seq

ChIP was performed as previously described^38^ with 10 μg of anti-LSD1 (ab17721; Abcam). A no antibody IP control and total input control were included to ensure specific enrichment compared to total genomic DNA. The quality of ChIP DNA was assessed on a 2100 Bioanalyzer (Agilent). ChIP libraries were prepared with 10 ng of ChIP DNA using the TruSeq® ChIP Sample Preparation Kit (Illumina) at the Ramaciotti Centre for Gene Function Analysis, University of New South Wales, Sydney. ChIP DNA libraries were then sequenced on the Illumina NextSeq. 500 using a 75 bp high output single-read run.

### ChIP-seq analysis

Reads were adapter stripped (CutAdapt), mapped to Hg19 (bowtie2), and duplicates removed (Picard). BedGraph files were created from the first read using HTSEQ. Enriched regions were called against Total Input with the first read of the pair using HOMER (-fragLength 200 findPeaks -style histone -F 4 -size 300 -minDist 300) and then filtered so that each region had at least 12.2 normalised reads. HOMER was used to annotate and profile the regions, search for enriched motifs and ontology, count tags in the regions, and profile histone and other marks. HOMER was also used to peak call enriched PKC-θ regions from previously published PKC-θ ChIP-seq data^38^. Overlapping peaks were defined as those with any overlap. PCCs of binding were determined using the tag counts in merged regions from PKC-θ and LSD1 standardised to 300 bp. R was used to determine if the center of the regions occurred within given distances to gene transcription start sites (TSSs; obtained from Affymetrix annotation file na.35). Microarray expression and ChIP-seq data are deposited in the Gene Expression Omnibus (GEO) under accession number GSE104755.

### Kinase profiling on peptide microarrays

Active recombinant PKC-θ was provided to JPT Peptide Technologies (Berlin, Germany) for kinase profiling on peptide microarrays. Unmodified LSD1 peptides were chemoselectively immobilised on glass slides and incubated with kinase solution in the presence of g-³³P-ATP prior to high-resolution phosphorimaging. GenepixPro 7.2 and ArrayPro 4.0 spot recognition software packages were used for data acquisition and analysis. Peptide constructs that displayed a normalised mean signal equal to or greater than 2 SDs above the mean were considered positive for phosphorylation events. Excel, R, and Python were used to determine the statistical significance of sequences and phosphorylation events.

### MDA-MB-231 mouse xenografts

Five-week-old female nude mice were acquired from the Animal Resources Centre (Perth) and allowed to acclimatize for one week in the animal facility at the John Curtin School of Medical Research (JCSMR) before experimentation. All experimental procedures were performed in accordance with the guidelines and regulations accessed and approved by The Australian National University Animal Experimental Ethics Committee (Ethics ID A2014/30). MDA-MB-231 human breast carcinoma cells were injected subcutaneously into the right mammary gland (2 × 10^6^ cells in 1:1 PBS and BD Matrigel Matrix). Tumours were measured using external calipers and calculated using the modified ellipsoidal formula: ½ (*a*/*b* ^2^), where *a* = longest diameter and *b* = shortest diameter. Tumours were allowed to grow to around 50 mm^3^ before commencing treatments (around 15 days). All treatments were given by IP injections. Tumours were excised and collected in DMEM supplemented with 2.5% FCS. Tumours were then finely minced using a surgical blade and incubated at 37 °C for 1 hour in DMEM 2.5% FCS and collagenase type 4 (Worthington-Biochem) (1 mg of collagenase/1 g of tumour). Digested tumours were spun and resuspended in DMEM 2.5% FCS before being passed through a 0.2 µM filter and used for fluorescence microscopy as described earlier.

### Isolation of circulating tumour cells

Liquid biopsies were collected from 10 patients with ER^+^/PR^+^/HER2^−^ Stage IV MBC and had received any form of systemic therapy. Disease burden was assessed by standard of care monitoring (CT scans (RECIST 1.1), blood work, clinical symptoms/judgement). Liquid biopsies were pre-enriched using the RosetteSep™ method to isolate CTCs by employing the RosetteSep™ Human CD45 Depletion Kit (15162, Stemcell Technologies) to remove CD45^+^ cells and red blood cells, using density gradient centrifugation with SepMate™-15 (IVD) density gradient tubes (85420, Stemcell Technologies) and Lymphoprep™ density gradient medium (07861, Stemcell Technologies). Enriched CTC samples were then quantified on the DEPArray™ single cell isolation system using the manufacture’s protocol. Samples were stained with anti-APC-CD45 (555485; BD Biosciences), anti-FITC-pan-cytokeratin (130-080-101; Miltenyi Biotec) and anti-PE-vimentin (562337; BD Biosciences) to confirm the presence of CTCs and produce a CTC count per 7.5 ml of blood. Only CD45^−^/Cytokeratin^+^/Vimentin^+^/DAPI^+^ cells were considered as CTCs. Purified CTC samples were then either left untreated or treated with either 3 mM pargyline or 500 µM phenelzine for 12 hours and then processed for high resolution immunofluorescence through methods described earlier. All experimental procedures relating to human studies were performed in accordance with the guidelines and regulations approved by the ACT Health Research Ethics and Governance Office: Human Research Ethics Committee, Building 10, The Canberra Hospital, Garran, ACT, 2605 (Ethics ID ETH.11.15.217). Written informed consent was received from all patients prior to inclusion in the study.

### Statistics

All comparisons between samples were evaluated using the two-tailed non-parametric Mann-Whitney test (GraphPad Prism) unless otherwise stated in figure legends. Where applicable, statistical significance is denoted by * for P ≤ 0.05, ** for P ≤ 0.01, *** for P ≤ 0.001, and **** for P ≤ 0.0001. Data are expressed as mean ± SE.

## Electronic supplementary material

Supplemental Information and Data
