# Author Correction: LSD1 activation promotes inducible EMT programs and modulates the tumour microenvironment in breast cancer

**DOI:** 10.1038/s41598-019-55020-1

**Published:** 2019-12-05

**Authors:** T. Boulding, R. D. McCuaig, A. Tan, K. Hardy, F. Wu, J. Dunn, M. Kalimutho, C. R. Sutton, J. K. Forwood, A. G. Bert, G. J. Goodall, L. Malik, D. Yip, J. E. Dahlstrom, A. Zafar, K. K. Khanna, S. Rao

**Affiliations:** 1 Health Research Institute, Faculty of ESTeM, University of Canberra, Bruce, ACT 2617 Australia; 2 QIMR Berghofer Medical Research Institute, 300 Herston Road, Herston, QLD 4006 Australia; 3 School of Biomedical Sciences, Charles Sturt University, Wagga Wagga, NSW 2678 Australia; 4 Gene Regulation Section, Centre for Cancer Biology, SA Pathology, Adelaide, SA 5000 Australia; 5 Department of Medical Oncology, The Canberra Hospital, Garran, ACT 2605 Australia; 6 ANU Medical School, Australian National University, Acton, ACT 2601 Australia; 7 Department of Anatomical Pathology, ACT Pathology, The Canberra Hospital, Garran, ACT 2605 Australia

Correction to: *Scientific Reports* 10.1038/s41598-017-17913-x, published online 08 January 2018

In this Article, the authors neglected to include some potential conflicts of interest. The Competing Interests section should read:

“In accordance with NHMRC policy and our ethical obligations as researchers, we report that the University of Canberra, Rao, McCuaig, Tan and Zafar have a financial interest in EpiAxis Therapeutics Pty Ltd and Forwood receive funding from EpiAxis Therapeutics Pty Ltd. Rao is also Chief Scientific Officer of EpiAxis Therapeutics Pty Ltd. We also have in place a plan for managing any potential conflicts arising from that involvement.”

