# Supplementary material for: LSD1 activation promotes inducible EMT programs and modulates the tumour microenvironment in breast cancer
Source: Sci Rep. 2018 Jan 8;8:73. doi: 10.1038/s41598-017-17913-x (PMC5758711; doi:10.1038/s41598-017-17913-x)
Supplement: Supplementary file 1 — Supplemental Information and Data [file 41598_2017_17913_MOESM1_ESM.pdf]

**LSD1 activation promotes inducible EMT programs and modulates the tumour microenvironment in breast cancer**

T. Boulding[1], R. D. McCuaig[1], A. Tan[1], K. Hardy[1], F. Wu[1], J. Dunn[1], M. Kalimutho[2], C. R. Sutton[1], J.K. Forwood[3], A.G. Bert[7], G. J. Goodall[7], L. Malik[4,5], D. Yip[4,5], J. E. Dahlstrom[5,6], A. Zafar[1], KK. Khanna[2] and S. Rao[1,*]

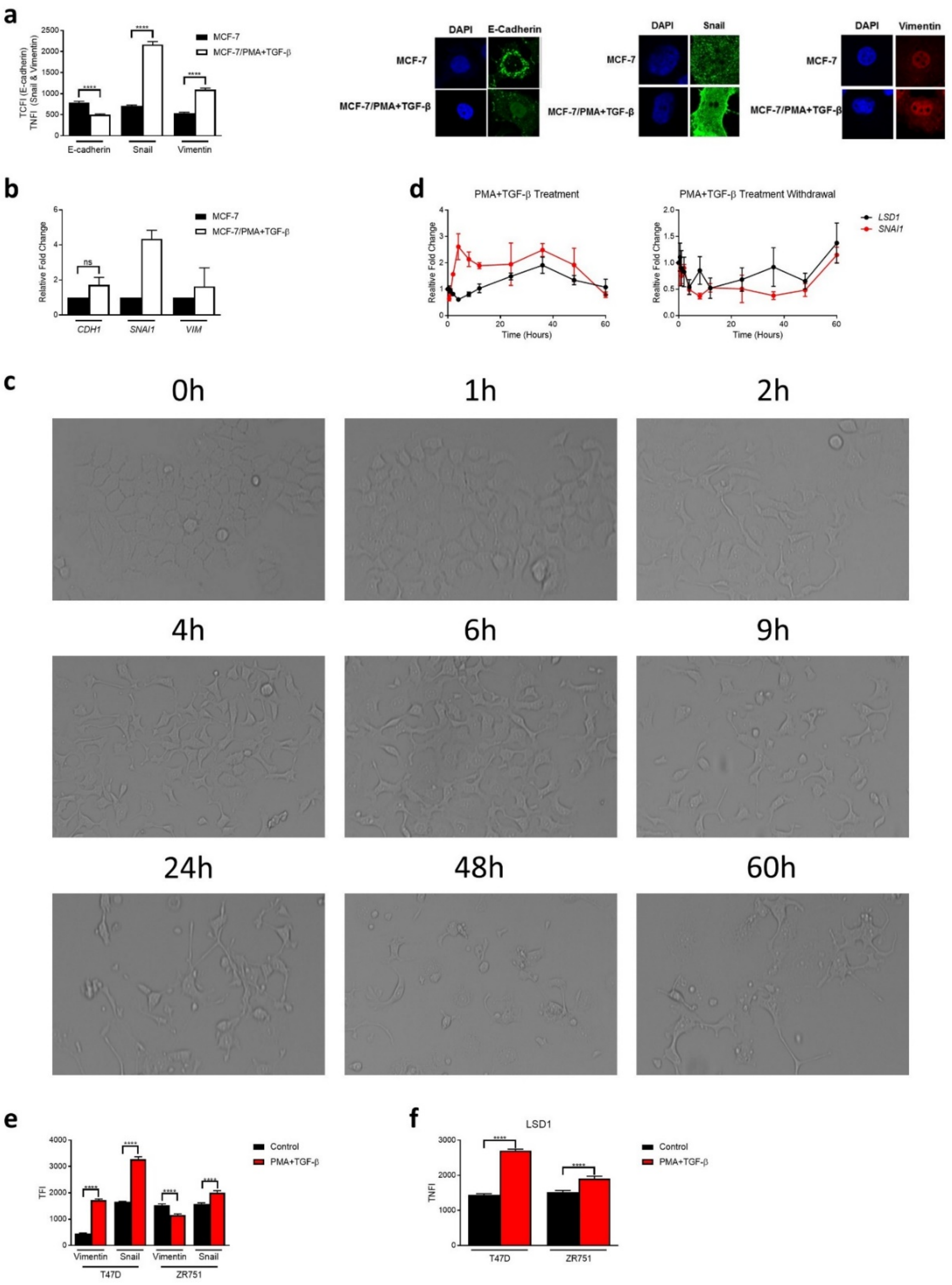

**Supplementary Figure 1**. Validation of EMT induction. (**a**) Immunofluorescence microscopy was performed on MCF-7 and MCF-7/PMA+TGF-β cells. Cells were fixed and probed with anti-E-cadherin, anti-Snail, and anti-vimentin antibodies. Graph depicts TCFI of E-cadherin and TNFI of Snail and vimentin for at least 30 individual cells ± SE. (**b**) qPCR was performed on MCF-7 and MCF-7/PMA+TGF-β cells and mRNA transcript levels for *CDH1*, *SNAI1* and *VIM* ± SE. (**c**) Phase-contrast microscopy was performed on MCF-7 cells after incubation with PMA+TGF-β at indicated time points. (**d**) *LSD1* and *SNAI1* transcript levels measured by qPCR in MCF-7 cells after incubation with PMA+TGF-β or withdrawal of PMA+TGF-β after 60 h incubation at the indicated time points. Data are expressed as fold change relative to 0 h stimulation or 0 h stimulation withdrawal (n = 3). Immunofluorescence microscopy was performed on T47D and ZR751 cells treated with PMA+TGF-β. (**e**) Cells were fixed and probed with anti-vimentin, and anti-Snail antibodies; or (**f**) anti-LSD1. Graphs depict the TNFI of vimentin, Snail or LSD1 for 20 individual cells ± SE.

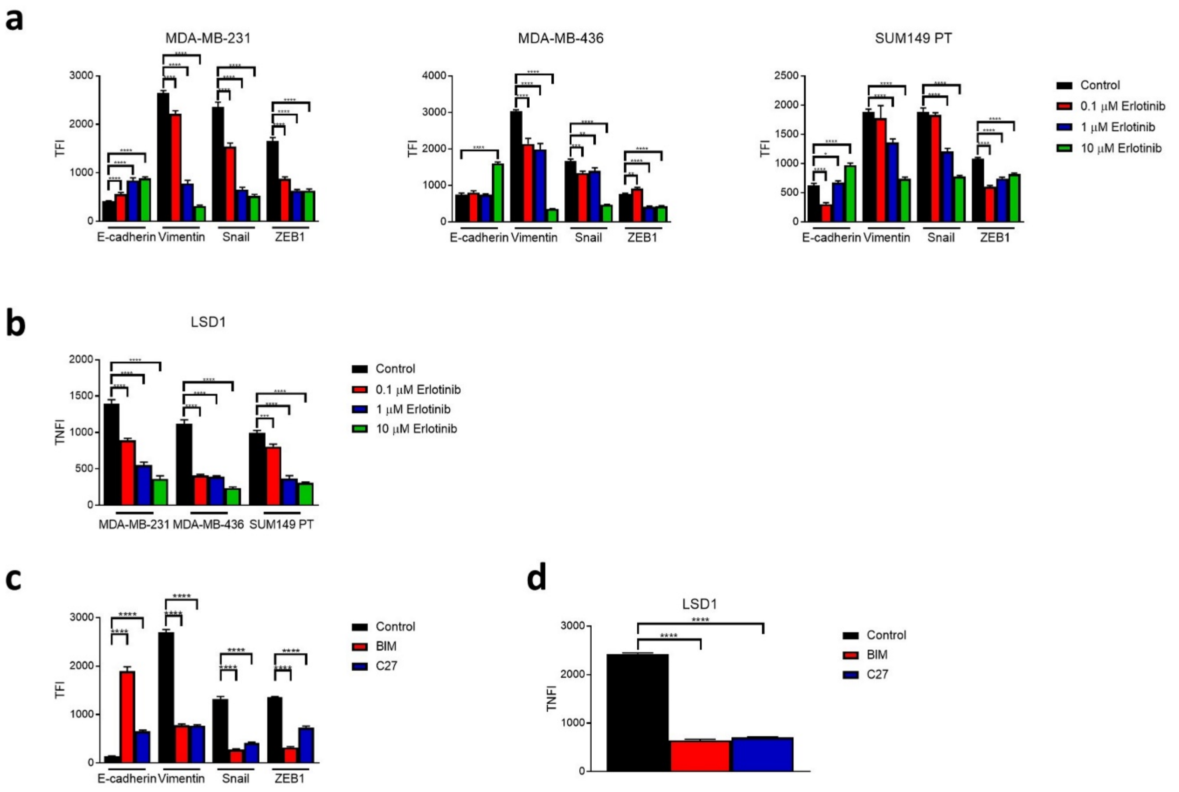

**Supplementary Figure 2**. Validation of MET induction. Immunofluorescence microscopy was performed on MDA-MB-231, MDA-MB-436 and SUM149 PT cells treated with indicated concentrations of erlotinib. (**a**) Cells were fixed and probed with anti-e-cadherin, anti-vimentin, anti-Snail antibodies and anti-ZEB1; or (**b**) anti-LSD1. Immunofluorescence microscopy was performed on MDA-MB-231 cells treated with BIM or C27. (**c**) Cells were fixed and probed with anti-e-cadherin, anti-vimentin, anti-Snail antibodies and anti-ZEB1; or (**d**) anti-LSD1. Graphs depict the TCFI of E-cadherin and the TNFI of vimentin, Snail or LSD1 for 20 individual cells ± SE.

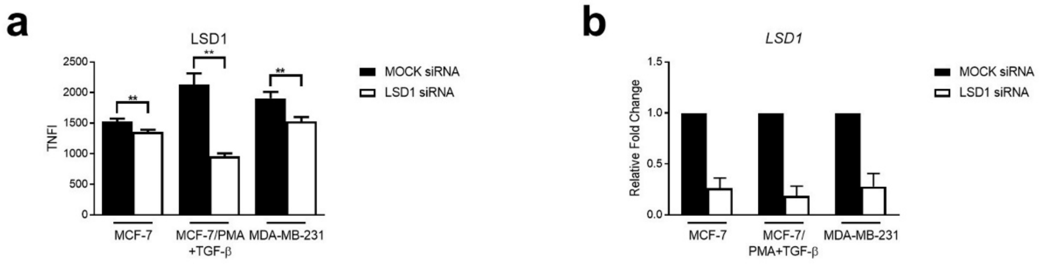


**Supplementary Figure 3**. Validation of siRNA-mediated knockdown of LSD1. (**a**) TNFI of LSD1 following immunofluorescence microscopy on mock and LSD1 siRNA treated MCF-7, MCF-7/PMA+TGF-β, and MDA-MB-231 cells fixed and probed with anti-LSD1 ± SE. (**b**) LSD1 mRNA transcript levels as measured by qPCR in MCF-7, MCF-7/PMA+TGF-β, and MDA-MB-231 cells after treatment with mock or LSD1 siRNA ± SE.

a

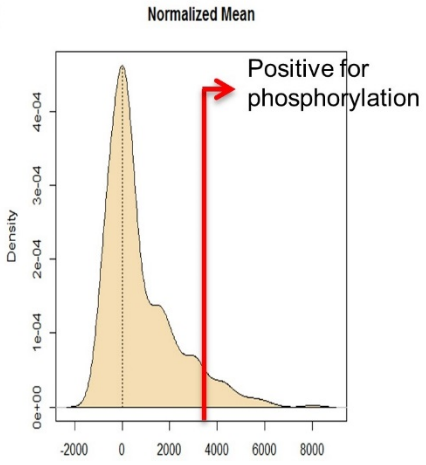


b

| | Top Ten Peptides positive for phosphorylation | Mean Signal |
|---|---|---|
| 1 | RTSRRKRAKVEYREM | 55353 |
| 2 | PEGRRTSRRKRAKVE | 54637 |
| 3 | LLEATSYLSHQLDFN | 54433 |
| 4 | DFEFTGSHLTVRNGY | 54045 |
| 5 | ESLANLSEDEYYSEE | 53964 |
| 6 | IAETPEGRRTSRRKR | 53869 |
| 7 | HSYLERHGLINFGIY | 53679 |
| 8 | VGGRVATFRKGNYVA | 53672 |
| 9 | YSYVAAGSSGNDYDL | 53492 |
| 10 | PYNSDTVLVHRVHSY | 53416 |

c

1
MLSGKKAAAA AAAAAAAATG TEAGPGTAGG SENGSEVAAQ PAGLSGPAEV
51
GPGAVGERTP RKKEPPRASP PGGLAEPPGS AGPQAGPTVV PGSATPMETG
101
IAETPEGRRT SRRKRAKVEY REMDESLANL SEDEYYSEEE RAKAEKEKK
151
LPPPPPQAPP EEENESEPEE PSGVEGAAFQ SRLPHDRMTS QEAACFPDII
201
SGPQQTQKVF LFIRNRTLQL WLDNPKIQLT FEATLQQLEA PYNSDTVLVH
251
RVHSYLERHG LINFGIYKRI KPLPTKKTGK VIIIGSGVSG LAAARQLQSF
301
GMDVTLLEAR DRVGGRVATF RKGNYVADLG AMVVTGLGGN PMAVVSKQVN
351
MELAKIKQKC PLYEANGQAV PKEKDEMVEQ EFNRLLEATS YLSHQLDFNV
401
LNNKPVSLGQ ALEVVIQLQE KHVKDEQIEH WKKIVKTQEE LKELLNKMVN
451
LKEKIKELHQ QYKEASEVKP PRDITAEFLV KSKHRDLTAL CKEYDELAET
501
QGKLEEKLQE LEANPPSDVY LSSRDRQILD WHFANLEFAN ATPLSTLSLK
551
HWDQDDDFEF TGSHLTVRNG YSCVPVALAE GLDIKLNTAV RQVRYTASGC
601
EVIAVNTRST SQTFIYKCDA VLCTLPLGVL KQQPPAVQFV PPLPEWKTSA
651
VQRMGFGNLN KVVLCFDRVF WDPSVNLFGH VGSTTASRGE LFLFWNLYKA
701
PILLALVAGE AAGIMENISD DVIVGRCLAI LKGIFGSSAV PQPKETVVSR
751
WRADPWARGS YSYVAAGSSG NDYDLMAQPI TPGPSIPGAP QPIPRLFFAG
801
EHTIRNYPAT VHGALLSGLR EAGRIADQFL GAMYTLPRQA TPGVPAQQSP

SM

**Supplementary Figure 4.** Top ten peptides positive for LSD1 phosphorylation. (**a**) Graph indicates the distribution of LSD1 peptide phosphorylation. The red bar indicates any events considered positive for phosphorylation (2 x SD above the mean). (**b**) Table indicates the sequence for the top ten peptides positive for phosphorylation and their mean signal intensity. (**c**) LSD1 amino acid sequence indicating the location of the top ten peptides positive for

phosphorylation. Red amino acids denote the NLS region, green bars denote the location of peptides and are numbered in order of mean signal intensity.

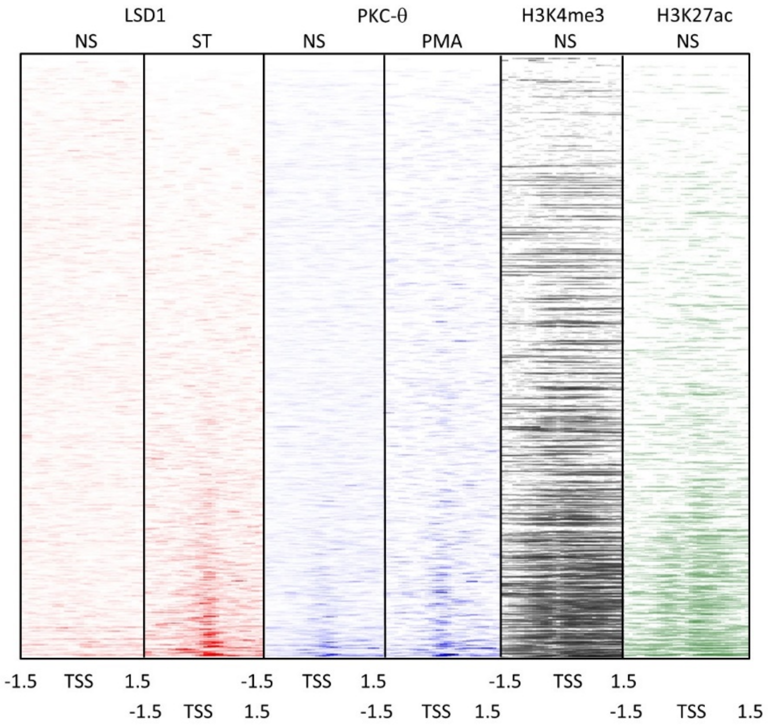


**Supplementary Figure 5**. Profile of LSD1, PKC-θ, H3K4me3 and H3K27ac around LSD1-sensitive genes. LSD1, PKC-θ, H3K4me3, and H3K27ac levels around the TSS of LSD1-sensitive genes. Reads are binned by 0.1 kb ± 0.5 kb around the TSS and are ranked by level of LSD1 within ± 1.5 kb of TSS.

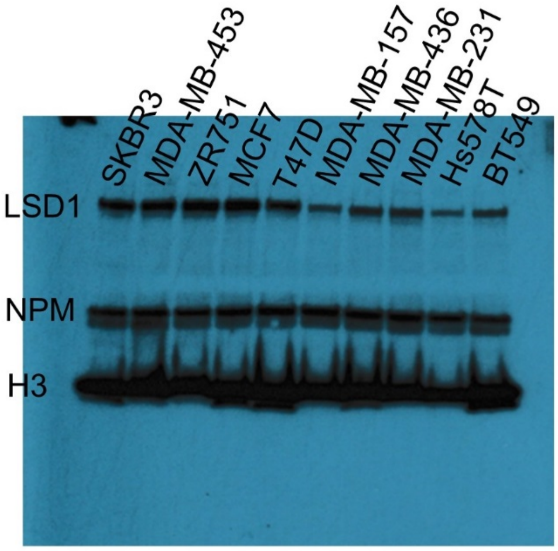


**Supplementary Figure 6**. Uncropped breast cancer cell line western blot. Uncropped western blots of indicated breast cancer cell line total cell extracts probed for LSD1. NPM and histone H3 were loading controls. All samples and antibodies were processed and run on the same gel.

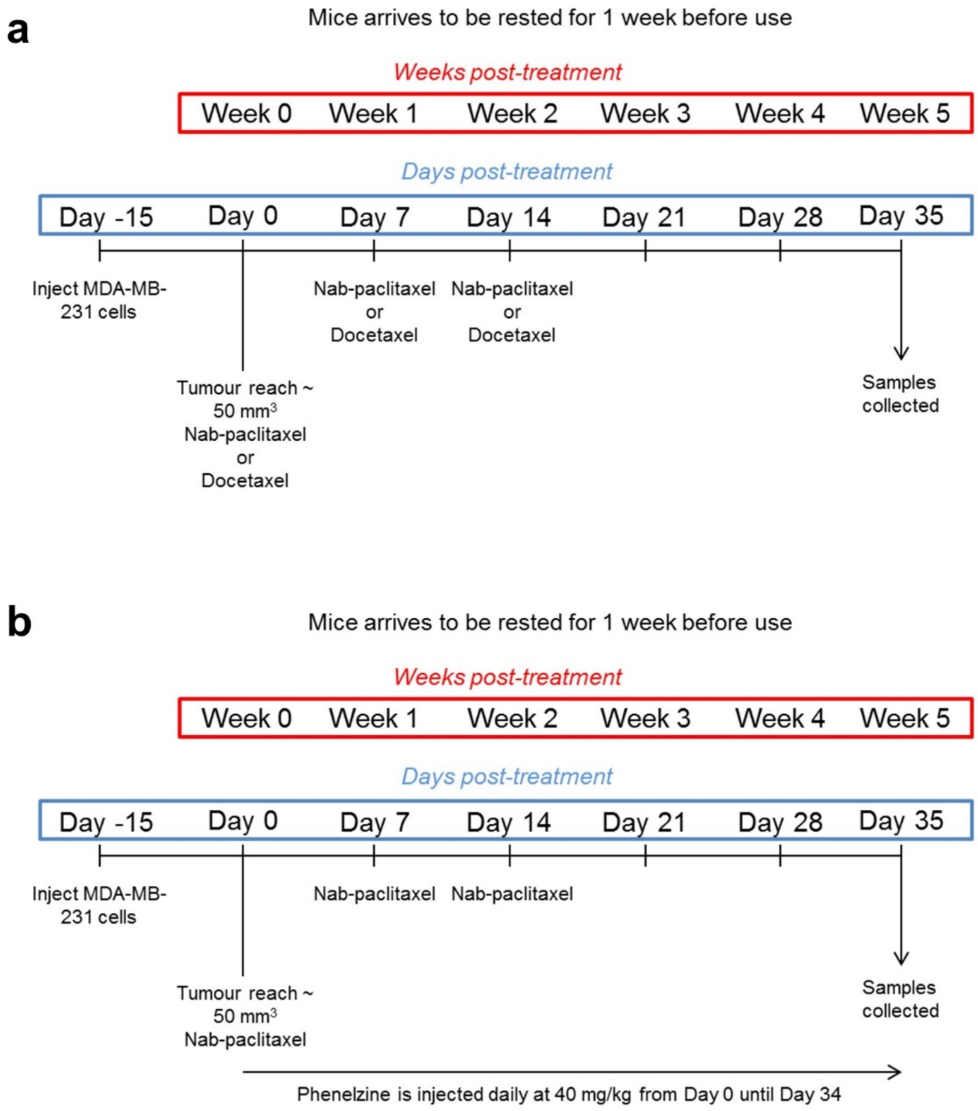


**Supplementary Figure 7.** Schematic of *in vivo* treatment regimens. Treatment regimens for *in vivo* experiments described relating to (**a**) Figure 5; or (**b**) Figure 6.

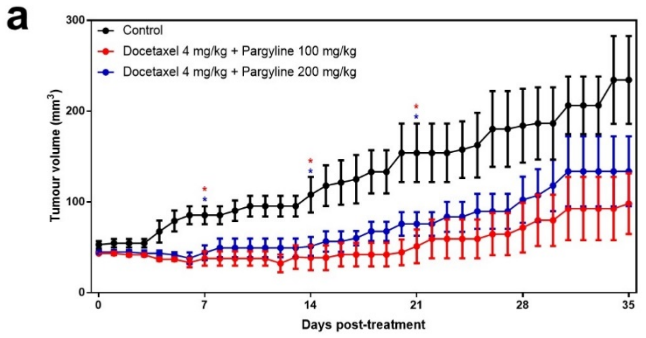


**Supplementary Figure 8.** Tumor growth after docetaxel and pargyline combination treatment. Tumor growth ($mm^3$) up to 5 weeks post-treatment with indicated treatments. Colored asterisk (*) denotes significance at that time point.

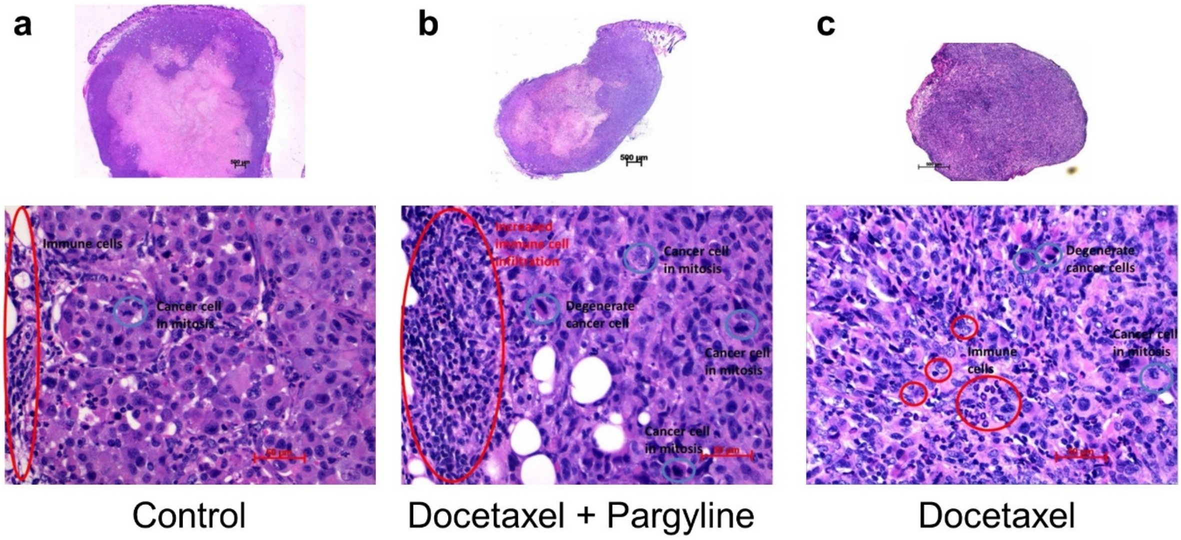


**Supplementary Figure 9.** H&E staining of control, docetaxel and combined pargyline-docetaxel-treated xenografts. Haematoxylin and eosin stained sections (original magnification x 400) from: (**a**) control; (**b**) docetaxel and pargyline combination; and (**c**) docetaxel alone treated mice. Scale bars = 50 µM.
